# Ki67 and breast cancer mortality in women with invasive breast cancer

**DOI:** 10.1093/jncics/pkad054

**Published:** 2023-08-11

**Authors:** Jake Probert, David Dodwell, John Broggio, Jackie Charman, Mitch Dowsett, Amanda Kerr, Paul McGale, Carolyn Taylor, Sarah C Darby, Gurdeep S Mannu

**Affiliations:** Nuffield Department of Population Health, University of Oxford, Oxford, UK; Nuffield Department of Population Health, University of Oxford, Oxford, UK; The National Disease Registration Service, NHS England, Leeds, UK; The National Disease Registration Service, NHS England, Leeds, UK; The Institute of Cancer Research, London, UK; Nuffield Department of Population Health, University of Oxford, Oxford, UK; Nuffield Department of Population Health, University of Oxford, Oxford, UK; Nuffield Department of Population Health, University of Oxford, Oxford, UK; Nuffield Department of Population Health, University of Oxford, Oxford, UK; Nuffield Department of Population Health, University of Oxford, Oxford, UK; Nuffield Department of Surgical Sciences, University of Oxford, Oxford, UK

## Abstract

**Background:**

The percentage of cells staining positive for Ki67 is sometimes used for decision-making in patients with early invasive breast cancer (IBC). However, there is uncertainty regarding the most appropriate Ki67 cut points and the influence of interlaboratory measurement variability. We examined the relationship between breast cancer mortality and Ki67 both before and after accounting for interlaboratory variability and 8 patient and tumor characteristics.

**Methods:**

A multicenter cohort study of women with early IBC diagnosed during 2009-2016 in more than 20 NHS hospitals in England and followed until December 31, 2020.

**Results:**

Ki67 was strongly prognostic of breast cancer mortality in 8212 women with estrogen receptor (ER)–positive, human epidermal growth factor receptor 2 (HER2)–negative early IBC (*P*_trend_ < .001). This relationship remained strong after adjustment for patient and tumor characteristics (*P*_trend_ < .001). Standardization for interlaboratory variability did little to alter these results. For women with Ki67 scores of 0%-5%, 6%-10%, 11%-19%, and 20%-29% the corresponding 8-year adjusted cumulative breast cancer mortality risks were 3.3% (95% confidence interval [CI] = 2.8% to 4.0%), 3.7% (95% CI = 3.0% to 4.4%), 3.4% (95% CI = 2.8% to 4.1%), and 3.4% (95% CI = 2.8% to 4.1%), whereas for women with Ki67 scores of 30%-39% and 40%-100%, these risks were higher, at 5.1% (95% CI = 4.3% to 6.2%) and 7.7% (95% CI = 6.6% to 9.1) (*P*_trend_ < .001). Similar results were obtained when the adjusted analysis was repeated with omission of pathological information about tumor size and nodal involvement, which would not be available preoperatively for patients being considered for neoadjuvant therapy.

**Conclusion:**

Our findings confirm the prognostic value of Ki67 scores of 30% or more in women with ER-positive, HER2-negative early IBC, irrespective of interlaboratory variability. These results also suggest that Ki67 may be useful to aid decision-making in the neoadjuvant setting.

Ki67 has been of interest as a marker of cell proliferation in breast cancer research for several decades. Particular attention surrounds the potential of Ki67 to help estimate long-term outcomes in early-stage invasive disease and to predict responsiveness to chemotherapy or endocrine therapy (1,2). Higher Ki67 scores have consistently been associated with poorer outcomes (3), and because Ki67 score data are available prior to surgery, they might be of use in identifying patients who would benefit from neoadjuvant therapy, or to identify the optimal neoadjuvant regimen (4).

Uncertainty currently exists regarding the optimal cut points for Ki67 to delineate high- and low-risk individuals in treatment decisions. The International Ki67 in Breast Cancer Working Group (IKWG) consensus recommended that Ki67 scores of 5% or less and 30% or more (but not 6%-29%) can be used for clinical decision making (2), whereas the widely used Predict online breast cancer decision aid (http://www.predict.nhs.uk) uses a 10% cutoff (5) and, for adjuvant abemaciclib treatment, a 20% cutoff has determined eligibility (6,7). There is also uncertainty regarding the prognostic potential of Ki67 in the neoadjuvant setting and the degree to which Ki67 scores provide additional information over and above established prognostic factors such as stage, grade and estrogen receptor (ER), progesterone receptor (PR), and human epidermal growth factor receptor 2 (HER2) status (8). Finally, there are concerns about the lack of reproducibility of Ki67 measurements across different pathology laboratories (1).

In England, Ki67 has been measured in specific hospitals and in certain circumstances, such as for patients enrolled in some trials. Therefore, to provide further information on the prognostic value of Ki67 for breast cancer mortality, we investigated optimal Ki67 cutoff values and explored interlaboratory variation in clinical use. We undertook a study of all women with early invasive breast cancer (IBC) in England for whom Ki67 had been measured at, or shortly after, their cancer diagnosis and we examined the association between Ki67 score and breast cancer mortality in different molecular subtypes.

## Methods

### Study population

Depersonalized data were obtained from the National Disease Registration Service (NDRS) on all 308 680 women registered in England during January 2009 to December 2016 with breast cancer as their first invasive cancer. The data included information on date of diagnosis, age at diagnosis, screening status, pathological staging (tumor size and number of positive nodes), grade, ER status, HER2 status, PR status, deprivation index, self-reported ethnicity, and, where applicable, date of emigration or death and cause of death, up to December 31, 2020. Ki67 scores were defined as the percentage of positively stained tumor cells among all the malignant cells assessed. Ki67 is not one of the standard variables listed in the NDRS data dictionary. However, when Ki67 has been measured, the result is included in text form in the pathology record. These records were provided to the study team, and information on Ki67 score was extracted using text interpretation programs. Tabulations of the sources of the Ki67 scores were provided by NDRS (Supplementary Table 1, available online).

Ki67 scores were included if their date was between 3 months before and 1 year after the cancer registry’s diagnosis date. In total, 20 457 such women were identified, of whom 6244 were then excluded for the following reasons: registration from a death certificate only, less than 3 months of follow-up, histology not IBC, younger than 18 years or older than 90 years at diagnosis; second cancer diagnosed within 3 months of first diagnosis, metastatic disease at diagnosis, or received neoadjuvant therapy. A further 1054 patients were excluded because the laboratory reporting the Ki67 measurement had reported fewer than 20 measurements in that year. After these exclusions, a total of 13 159 women remained in the study (Supplementary Figure 1, available online).

### Method of analysis

Ki67 scores were grouped according to the cut points specified in the IKWG (5% and 30%), in the PREDICT online tool (10%), and in the abemaciclib treatment guidelines (20%). However, nearly half of the breast cancer deaths were in women with Ki67 scores of 30% or more. Therefore, an additional cut point at 40% was introduced, resulting in 6 categories: 0%-5%, 6%-10%, 11%-19%, 20%-29%, 30%-39%, and 40%-100%.

Women contributed to the person-years from 3 months after their breast cancer diagnosis until the earliest of death, emigration, 95th birthday, or December 31, 2020. Crude annual breast cancer mortality rates were estimated by dividing the number of deaths by the number of person-years. Adjusted mortality rates and rate ratios were estimated using Poisson regression by including the number of person-years as the exposure and all of the available characteristics as explanatory variables apart from the use of chemotherapy, which might otherwise have been a source of confounding bias. These analyses were repeated separately for women with and without a record of receiving chemotherapy. Adjusted mortality rates were re-estimated after pathological tumor size and number of positive nodes were omitted from the adjustment, as these factors would not be available for women being considered for neoadjuvant therapy (clinical staging was not available). Adjusted cumulative breast cancer mortality risks were derived from the adjusted mortality rates. Further details are in sections A-C of the Supplementary Methods (available online).

To investigate interlaboratory variation, the Ki67 scores were transformed using natural logarithms. A laboratory-dependent constant was then added to each transformed score, so that the mean score was the same in each laboratory. The equality of Ki67 distributions in different laboratories was assessed using Kruskal-Wallis rank tests. Further details are in section D of Supplementary Methods and Supplementary Figure 2 (available online). Finally, as it was observed that there was considerable digit preference in the recording of Ki67 scores, the analyses were repeated using cut points that avoided the preferred digits. Further details are in section E of Supplementary Methods (available online). All calculations were performed using Stata version 17.1. All statistical tests were 2-sided and a 5% cutoff for statistical significance was used.

### Ethical approval

This study was approved by Public Health England’s Office for Data Release (reference ODR1718_390). Informed consent from individual participants was not required.

## Results

### Characteristics of the study population

**Figure 1.**
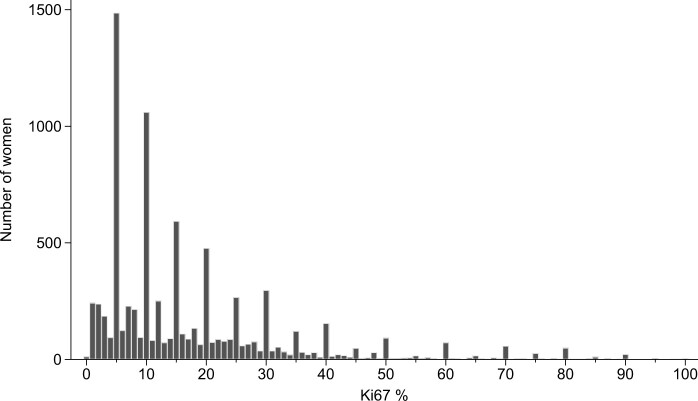
Distribution of Ki67 score percentages in 8212 women with ER-positive and HER2-negative breast cancer. ER = estrogen receptor; HER2 = human epidermal growth factor receptor 2.

Among the 13 159 women with early IBC and Ki67 measured at or shortly after diagnosis, 8212 (62.4%) women had ER-positive, HER2-negative disease (Figure 1). The median Ki67 score was 12% (Table 1). Women with scores of 30% or above were likely to be younger at diagnosis; not to be diagnosed through breast screening; have larger tumors, node-positive disease, high-grade disease, negative PR status, non-White ethnicity; and to have received chemotherapy than those with lower scores. The proportion of women with scores of 30% or above varied little with calendar period of diagnosis, and index of multiple deprivation was not associated with Ki67 score. During a median follow-up of 6.2 years, 760 women died, including 263 deaths from breast cancer. Results for other molecular subtypes are given in section F of the Supplementary Methods, Supplementary Table 2, and Supplementary Figures 3-5 (available online).

**Table 1.** Distribution of women with ER-positive and HER2-negative breast cancer by percentage Ki67 score and patient and tumor characteristics^a^

Characteristic	Ki67 score (%)						Total No. of women	Total No. of breast cancer deaths
	[0-5]	[6-10]	[11-19]	[20-29]	[30-39]	[40-100]		
Calendar period of diagnosis (*P* = .005)								
2009-2012	453 (31)	319 (22)	221 (15)	224 (15)	104 (7)	153 (10)	1474 (100)	71
2013-2014	901 (28)	706 (21)	596 (18)	508 (15)	264 (8)	317 (10)	3292 (100)	99
2015-2016	904 (27)	698 (20)	667 (19)	572 (17)	284 (8)	321 (9)	3446 (100)	93
Age at diagnosis, y (*P* < .001)								
18-39	35 (14)	41 (16)	34 (14)	34 (14)	36 (14)	71 (28)	251 (100)	20
40-49	320 (22)	291 (21)	218 (16)	230 (17)	125 (9)	202 (15)	1386 (100)	50
50-64	1046 (31)	734 (21)	633 (18)	537 (15)	260 (7)	287 (8)	3497 (100)	64
65-70	460 (30)	325 (21)	316 (20)	227 (15)	108 (7)	115 (7)	1551 (100)	44
71-79	301 (27)	237 (21)	209 (19)	198 (18)	87 (8)	75 (7)	1107 (100)	53
80-89	96 (21)	95 (23)	74 (18)	78 (19)	36 (9)	41 (10)	420 (100)	32
Screen-detected cancer (*P* < .001)								
Screen detected	1077 (33)	732 (22)	661 (20)	468 (14)	182 (6)	176 (5)	3296 (100)	37
Not screen detected	1181 (23)	991 (20)	823 (17)	836 (17)	470 (10)	615 (13)	4916 (100)	226
Tumor size, mm (*P* < .001)								
1-20	1482 (30)	1111 (23)	958 (20)	752 (15)	292 (6)	282 (6)	4877 (100)	62
21-50	516 (22)	430 (18)	357 (15)	411 (17)	282 (12)	386 (16)	2382 (100)	146
>50	71 (22)	49 (15)	57 (17)	60 (18)	41 (12)	52 (16)	330 (100)	30
Unknown	189 (31)	133 (21)	112 (18)	81 (13)	37 (6)	71 (11)	623 (100)	25
No. of positive nodes (*P* < .001)								
0	1580 (30)	1191 (22)	992 (18)	823 (15)	369 (7)	439 (8)	5394 (100)	81
1-3	452 (24)	352 (19)	337 (18)	329 (18)	191 (10)	206 (11)	1867 (100)	79
4-9	54 (16)	51 (15)	55 (16)	78 (23)	38 (11)	64 (19)	340 (100)	44
≥10	25 (17)	18 (12)	24 (16)	19 (13)	26 (17)	37 (25)	149 (100)	40
Unknown	147 (32)	111 (24)	76 (16)	55 (12)	28 (6)	45 (10)	462 (100)	19
Tumor grade (*P* < .001)								
Low	740 (45)	444 (27)	287 (18)	114 (7)	32 (2)	18 (1)	1635 (100)	11
Medium	1442 (27)	1156 (23)	1054 (21)	895 (18)	324 (6)	235 (5)	5106 (100)	126
High	69 (6)	113 (8)	136 (9)	294 (20)	292 (20)	537 (37)	1441 (100)	125
Unknown	7 (25)	10 (33)	7 (23)	1 (3)	4 (13)	1 (3)	30 (100)	1
PR status (*P* < .001)								
Positive	1385 (25)	1176 (21)	1094 (20)	976 (17)	467 (8)	480 (9)	5578 (100)	145
Negative	162 (23)	120 (17)	108 (15)	104 (14)	61 (8)	163 (23)	718 (100)	60
Unknown	711 (37)	427 (22)	282 (15)	224 (12)	124 (6)	148 (8)	1916 (100)	58
Chemotherapy (*P* < .001)								
Yes	303 (16)	292 (14)	296 (14)	375 (18)	287 (14)	501 (24)	2054 (100)	131
Not recorded^b^	1955 (32)	1431 (23)	1188 (19)	929 (15)	365 (6)	290 (5)	6158 (100)	132
Index of multiple deprivation (*P* = .24)								
<20% (least deprived)	525 (30)	371 (21)	293 (17)	257 (15)	124 (7)	175 (10)	1745 (100)	49
20%-39%	459 (28)	343 (21)	298 (18)	254 (16)	130 (8)	153 (9)	1637 (100)	47
40%-59%	415 (29)	321 (21)	274 (18)	228 (15)	124 (8)	135 (9)	1497 (100)	50
60%-79%	391 (26)	328 (21)	296 (19)	247 (16)	132 (9)	137 (9)	1531 (100)	44
80+% (most deprived)	468 (25)	360 (20)	323 (18)	318 (18)	142 (8)	191 (11)	1802 (100)	73
Ethnicity (*P* < .001)								
Black	25 (14)	38 (21)	33 (19)	36 (20)	19 (11)	27 (15)	178 (100)	3
East Asian	5 (13)	8 (22)	7 (19)	6 (16)	3 (8)	8 (22)	37 (100)	2
Other^c^	28 (22)	22 (17)	24 (18)	23 (18)	17 (13)	16 (12)	130 (100)	8
South Asian	35 (20)	33 (18)	37 (20)	34 (19)	16 (9)	26 (14)	181 (100)	8
White	1956 (28)	1476 (21)	1266 (18)	1109 (16)	558 (8)	667 (9)	7032 (100)	229
Unknown	209 (32)	146 (22)	117 (18)	96 (15)	39 (6)	47 (7)	654 (100)	13
								
Total No. of women (%)	2258 (27)	1723 (21)	1484 (18)	1304 (16)	652 (8)	791 (10)	8212 (100)	—
Total No. of breast cancer deaths	37	36	30	36	36	88	—	263
								
Ki67, median (IQR)	5 (3-5)	10 (8-10)	15 (13-16)	23 (20-25)	31 (30-35)	50 (43-70)	12 (5-23)	—
Ki67, mean (range)	4 (0-5)	9 (6-10)	15 (11-19)	23 (20-29)	32 (30-39)	57 (40-100)	17 (0-100)	—

a Values are No. of women (%) unless otherwise indicated. *P* values are for χ^2^ tests of independence between characteristic and Ki67 score. IQR = interquartile range; PR = progesterone receptor.

b Unable to distinguish between chemotherapy that has not been recorded and not receiving chemotherapy.

c Other self-reported ethnicity.

### Breast cancer mortality and Ki67 scores in ER-positive, HER2-negative disease

**Figure 2.**
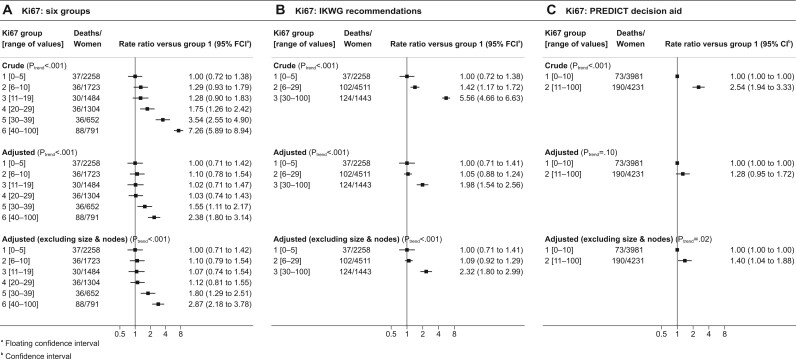
Breast cancer mortality rate ratios in women with ER-positive and HER2-negative breast cancer by percentage Ki67 score. **A)** Ki67 categorized in 6 groups. **B)** Ki67 grouped according to the IKWG recommendations. **C)** Ki67 grouped according to the PREDICT decision aid. Adjustment in the middle row is for all variables shown in Table 1 (except chemotherapy) using the categories shown in Table 1. Adjustment in the bottom row is similar but also omits tumor size and number of positive nodes. Breast cancer mortality rate ratios by other variables shown in Table 1 are in Supplementary Figures 6 and 7 (available online). Analyses of all-cause mortality are in Supplementary Figure 8 (available online). ER = estrogen receptor; HER2 = human epidermal growth factor receptor 2; IKWG = International Ki67 in Breast Cancer Working Group.

**Figure 3.**
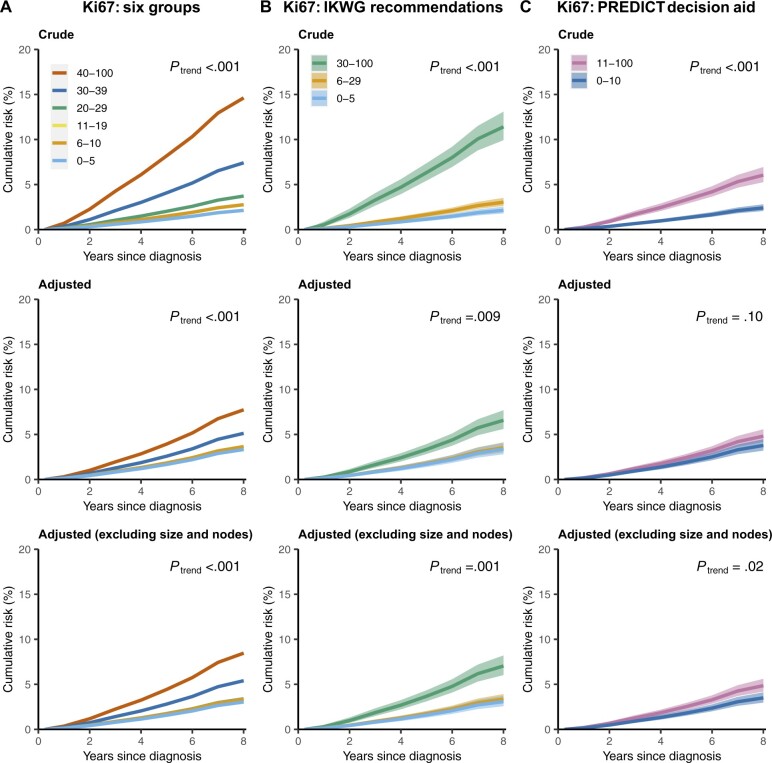
Cumulative breast cancer mortality risks in women with ER-positive and HER2-negative breast cancer by time since diagnosis and percentage Ki67 score. **A)** Ki67 categorized in 6 groups. **B)** Ki67 grouped according to the IKWG recommendations. **C)** Ki67 grouped according to the PREDICT decision aid. Top row based on crude rates, middle row based on rates adjusted for all other variables shown in Table 1 (except chemotherapy), bottom row based on rates adjusted for all other variables in Table 1 except chemotherapy, tumor size, and number of positive nodes. Shaded areas in **B)** and **C)** show 95% confidence intervals. Plotted values and numbers of women at risk are in Supplementary Tables 3, (i)-(iii) (available online). Analyses of all-cause mortality are in Supplementary Figure 9 (available online). ER = estrogen receptor; HER2 = human epidermal growth factor receptor 2; IKWG = International Ki67 in Breast Cancer Working Group.

In women with ER-positive, HER2-negative disease, the crude breast cancer mortality rate tended to increase with Ki67 score, with rate ratios of 1.00, 1.29, 1.28, 1.75, 3.54, and 7.26 for Ki67 scores of 0%-5%, 6%-10%, 11%-19%, 20%-29%, 30%-39%, and 40%-100%, respectively (*P*_trend_ < .001) (Figure 2, A, top panel and Supplementary Figures 6 and 7, available online). The 8-year cumulative breast cancer mortality risks increased correspondingly, taking values of 2.1% (95% confidence interval [CI] = 1.8% to 2.5%), 2.8% (95% CI = 2.3% to 3.3%), 2.8% (95% CI = 2.3% to 3.3%), 3.7% (95% CI = 3.1% to 4.4%), 7.4% (95% CI = 6.2% to 8.8%), and 14.6% (95% CI = 12.7% to 16.8%) (*P*_trend_ < .001) (Figure 3, A, top panel, and Supplementary Table 3, available online).

When the analysis was repeated after adjusting for all factors shown in Table 1, apart from chemotherapy, the breast cancer mortality rate ratios still tended to increase with increasing Ki67 score, but the increases were smaller than those in the crude analysis, taking values of 1.00, 1.10, 1.02, 1.03, 1.55, and 2.38 for Ki67 scores of 0%-5%, 6%-10%, 11%-19%, 20%-29%, 30%-39%, and 40%-100% respectively (*P*_trend_ < .001) (Figure 2, A, middle panel). The corresponding 8-year cumulative breast cancer mortality risks varied little over the first 4 categories, taking values of 3.3% (95% CI = 2.8% to 4.0%), 3.7% (95% CI = 3.0% to 4.4%), 3.4% (95% CI = 2.8% to 4.1%), and 3.4% (95% CI = 2.8% to 4.1%) for women with Ki67 scores of 0%-5%, 6%-10%, 11%-19%, and 20%-29%, respectively (Figure 3, A, middle panel). However, for women with Ki67 scores of 30%-39% and 40%-100%, the 8-year cumulative risks were higher, at 5.1% (95% CI = 4.3% to 6.2%) and 7.7% (95% CI = 6.6% to 9.1%), respectively. When the analysis was repeated adjusting for just 1 variable at a time, grade was the strongest confounding factor (Supplementary Table 4, available online).

**Figure 4.**
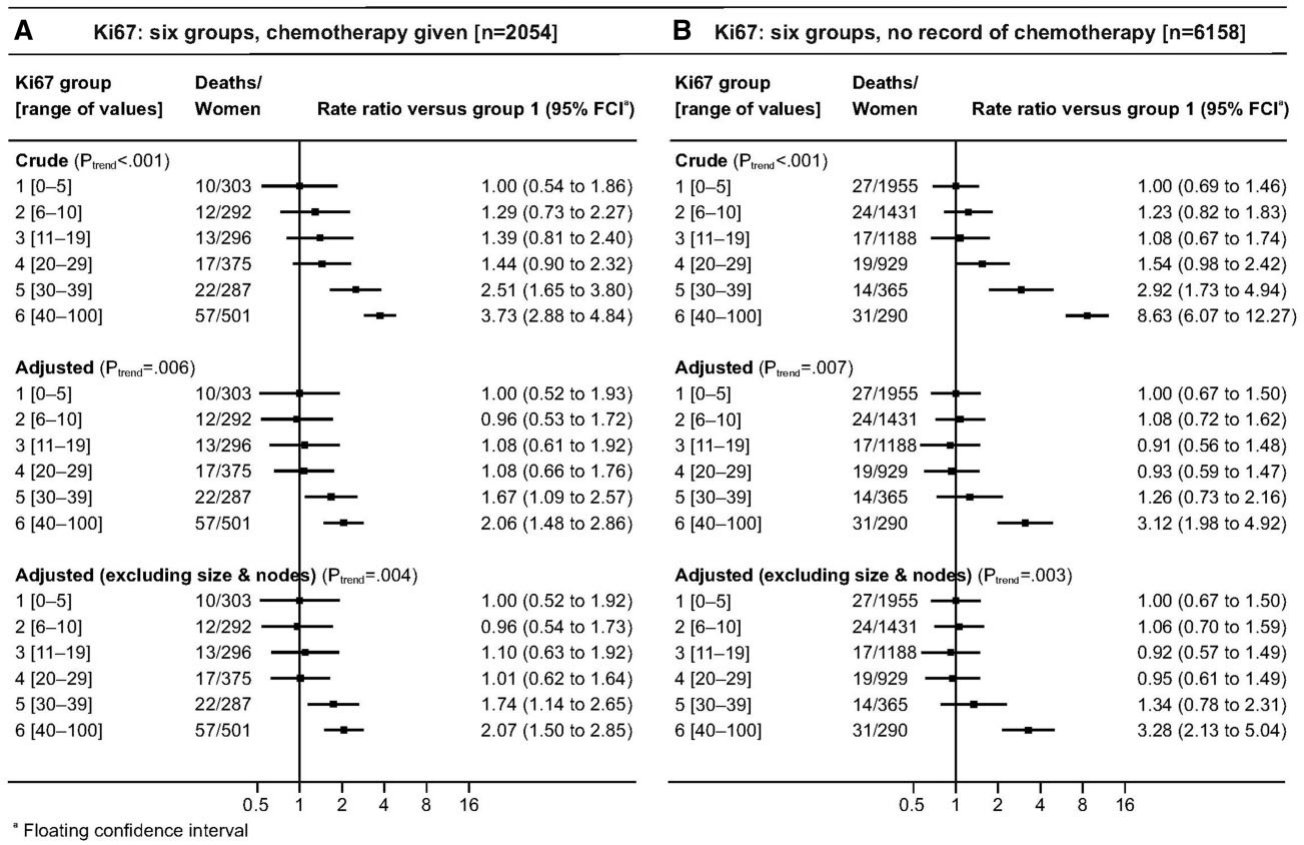
Breast cancer mortality rate ratios in women with ER-positive and HER2-negative breast cancer by percentage Ki67 score categorized in 6 groups: **A)** for women with and **B)** for women without a record of receiving chemotherapy treatment. Adjustment in middle row is for all other variables shown in Table 1 using the categories shown in Table 1 (except chemotherapy). Adjustment in bottom row is for all other variables shown in Table 1 except chemotherapy, tumor size and number of positive nodes. ER = estrogen receptor; HER2 = human epidermal growth factor receptor 2.

When the adjusted analysis was repeated with omission of pathological information on tumor size and nodal involvement, which would not be available for patients being considered for neoadjuvant therapy, results were similar to those obtained when these variables were included (Figure 2, A, and Figure 3, A, bottom panels). When all 3 analyses were conducted separately for women who received and who were not recorded to have received chemotherapy, the results in the 2 groups were similar (Figure 4). Trends were also similar when considering all-cause mortality (Supplementary Figures 8 and 9, available online).

### IKWG and PREDICT Ki67 cut points and breast cancer mortality

When the above analyses were repeated based solely on the 3 categories specified in the IKWG consensus, the crude breast cancer mortality rate ratios were 1.00, 1.42, and 5.56 for Ki67 scores of 0%-5%, 6%-29%, and 30%-100%, respectively (*P*_trend_ < .001, Figure 2, B, top panel). The corresponding 8-year cumulative breast cancer mortality risks were 2.1% (95% CI = 1.8% to 2.5%), 3.0% (95% CI = 2.6% to 3.5%) and 11.4% (95% CI = 9.9% to 13.1%) (Figure 3, B, top panel). With adjustment for all other factors shown in Table 1, apart from receipt of chemotherapy, the breast cancer mortality rate ratios were 1.00 and 1.05 for Ki67 scores of 0%-5% and 6%-29%, whereas for Ki67 scores of 30%-100% the rate ratio was 1.98 and the corresponding adjusted 8-year cumulative breast cancer mortality risks were 3.4% (95% CI = 2.8% to 4.1%), 3.5% (95% CI = 3.0% to 4.1%) and 6.6% (95% CI = 5.6% to 7.7%) (Figure 3, B, middle panel). Similar results were obtained when the adjusted analysis was repeated omitting pathological information about tumor size and nodal involvement (Figure 2, B, and Figure 3, B, bottom panel).

When the analysis was repeated using just the 2 PREDICT categories, the crude breast cancer mortality rates were 1.00 and 2.54 for Ki67 scores of 0%-10% and 11%-100%, respectively (*P*_trend_ < .001, Figure 2, C, top panel), leading to 8-year cumulative breast cancer mortality risks of 2.4% (95% CI = 2.1% to 2.8%) and 6.0% (95% CI = 5.3% to 6.9%), respectively (Figure 3, C, top panel). After full adjustment, these mortality rates were 1.00 and 1.28, and did not differ statistically significantly (*P*_trend_ = .10), while the adjusted 8-year cumulative breast cancer mortality risks were 3.8% (95% CI = 3.2% to 4.5%) and 4.8% (95% CI = 4.1% to 5.6%), respectively (Figure 3, C, top panel). When the adjusted analysis was repeated omitting pathological information on tumor size and nodal involvement, the rate ratios were 1.00 and 1.40 (*P*_trend_ = .02), corresponding to adjusted 8-year cumulative breast cancer mortality risks of 3.5% (95% CI = 3.0% to 4.1%) and 4.9% (95% CI = 4.2% to 5.6%) respectively (Figure 2, C, and Figure 3, C, bottom panel).

### Interlaboratory variation and digit preference

**Figure 5.**
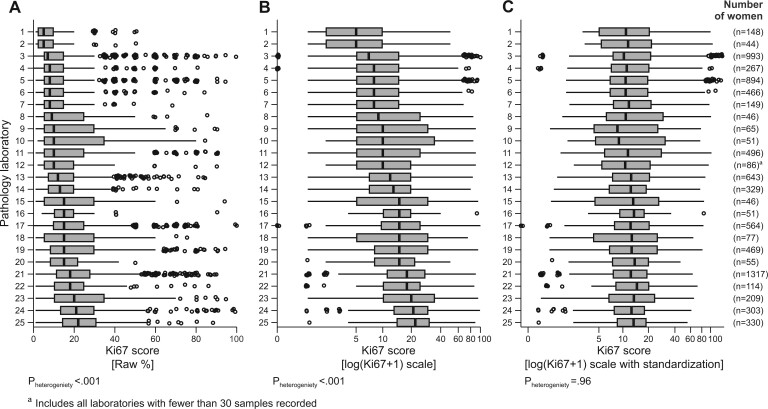
Box and whisker plots of Ki67 scores from 25 different pathology laboratories for 8212 women with ER-positive and HER2-negative breast cancer: **A)** raw scores, **B)** log-transformed scores and **C)** standardized Ki67 scores. The boxes include all values from the 25th to 75th centile (ie, the IQR), the vertical internal lines are the medians (laboratories are ordered by value of median in **A)**). This IQR determines the length of the whiskers. The whiskers extend 1.5 times the IQR above and below the 75th and 25th centile respectively. Dots represent outlier values that are outside the range of the whiskers. The number of samples that the laboratory recorded in the study is in the right-hand column. See section D of Supplementary Methods (available online) for further details on the standardization method. ER = estrogen receptor; HER2 = human epidermal growth factor receptor 2; IQR = interquartile range.

**Figure 6.**
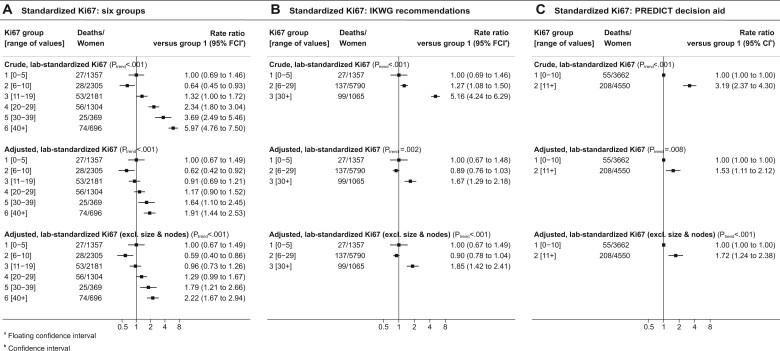
Breast cancer mortality rate ratios in women with ER-positive and HER2-negative breast cancer by percentage Ki67 score with standardization for pathology laboratory. **A)** Standardized Ki67 categorized in 6 groups, **B)** standardized Ki67 grouped according IKWG recommendations, **C)** standardized Ki67 grouped according to PREDICT decision aid. In middle row adjustment in middle panels is for all variables shown in Table 1 (except chemotherapy) using the categories shown in Table 1. In bottom row adjustment is for all variables except chemotherapy, tumor size and number of positive nodes. Breast cancer mortality rate ratios by other variables shown in Table 1 are in Supplementary Figures 10 and 11 (available online). ER = estrogen receptor; HER2 = human epidermal growth factor receptor 2; IKWG = International Ki67 in Breast Cancer Working Group.

The distributions of the Ki67 scores in the different laboratories were skewed with heavy upper tails (Figure 5, A, and Supplementary Figure 2, A, available online). However, with a logarithmic transformation [*y* = ln(*x* + 1)], the distributions were close to normal (Figure 5, B, Supplementary Figure 2, B, available online). A further laboratory-specific scalar shift on the logarithmic scale bringing the means together completed the standardization (Figure 5, C). The distributions of the standardized Ki67 scores in the different laboratories did not statistically significantly differ from each other (*P* = .96). When the analyses shown in Figure 2 were repeated using the standardized Ki67 scores, the prognostic value of Ki67 using either the IKWG or the PREDICT cut points was similar to that based on the unstandardized scores (Figure 6). Considering the 6 groups, the standardized scores of 6% and above were more strongly prognostic of breast cancer mortality than the unstandardized ones. However, scores of 5% and below were associated with larger breast cancer mortality rates relative to scores of 6%-10% after standardization than before it. The adjusted rate ratios for the other known characteristics were unchanged with standardization (Supplementary Figures 10 and 11, available online).

**Figure 7.**
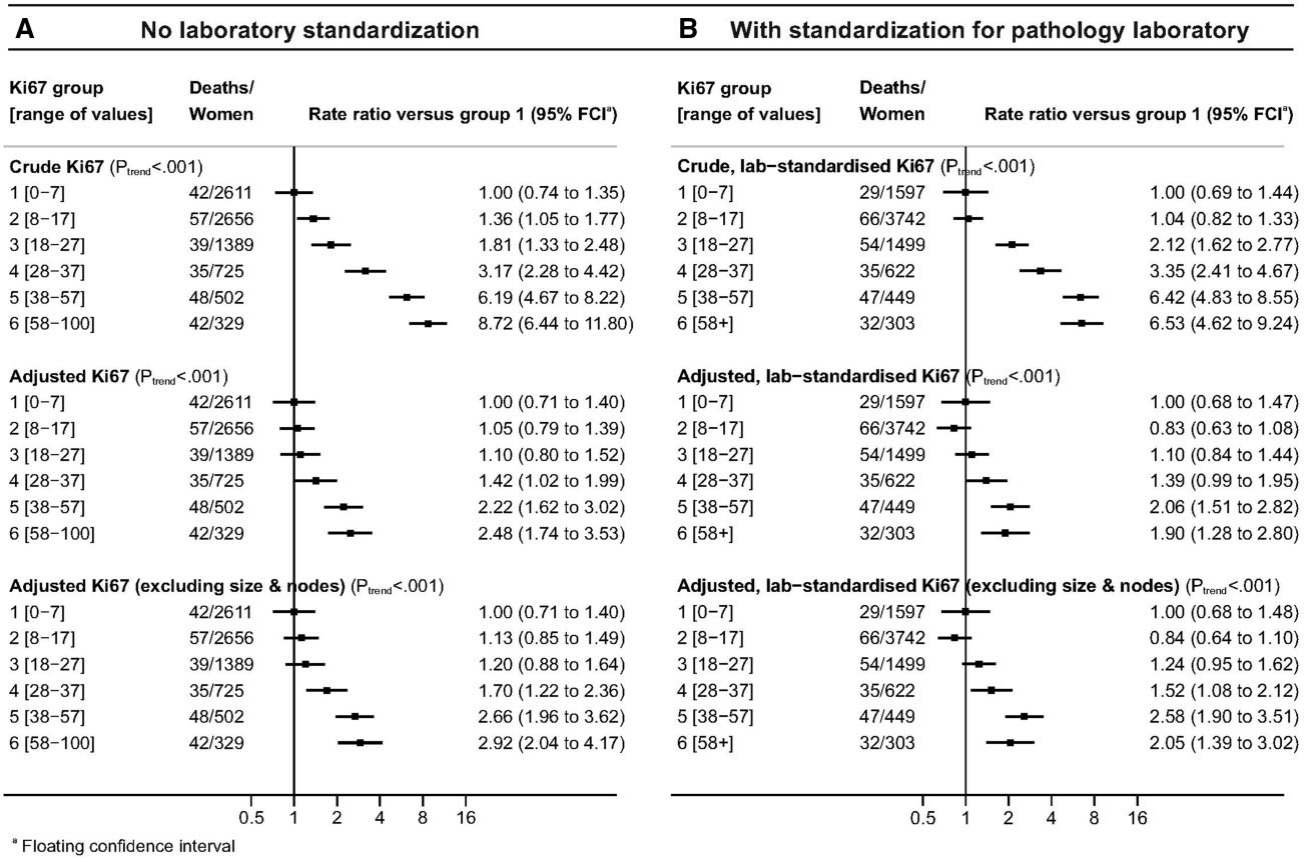
Breast cancer mortality rate ratios by Ki67 score classified into 6 groups avoiding the use of preferred digits as cut points in women with ER-positive and HER2-negative breast cancer: **A)** without standardization and **B)** with standardization for pathology laboratory. Adjustment in middle panels is for all variables shown in Table 1 using the categories shown in Table 1. Adjustment in bottom panel is for all variables except tumor size and number of positive nodes. ER = estrogen receptor; HER2 = human epidermal growth factor receptor 2.

The recorded Ki67 scores exhibited considerable digit preference, with most scores reported as 5, 10, 20, 30, etc, and relatively few reported as 4, 6, 9, 11, 19, 21, 29, 31, etc (Figure 1). This finding suggests that, for example, many women for whom a score of 5 is reported may actually have a score of 3, 4, 6, or 7. Therefore, to mitigate any effect of digit preference, we replaced the 6 categories of Ki67 by 6 categories that did not use the preferred digits as cut points: 0%-7%, 8%-17%, 18%-27%, 28%-37%, 38%-57%, and 58%-100%. Repeating the analyses shown in Figure 2, A, Figure 6, A, and Figure 4 using these alternative categories yielded broadly similar results, although the predictive power of Ki67 was increased (Figure 7 and Supplementary Figure 12, available online).

## Discussion

Our findings in this large multicenter study in National Health Service hospitals in England confirm the prognostic ability of the Ki67 score for breast cancer mortality in women with ER-positive, HER2-negative early IBC. After adjusting for a wide range of patient and tumor characteristics, Ki67 remained prognostic for Ki67 scores above about 30%, and strongly prognostic for scores above about 40%. These results changed little when pathological tumor size and extent of nodal involvement were omitted from the adjustment, suggesting that Ki67 may be useful in the neoadjuvant setting. These findings support the 30% Ki67 cut point suggested by the IKWG to denote high-risk tumors, more so than the 10% cut point used in the PREDICT decision aid. Last, although there was clear evidence of systematic differences between laboratories, standardization for interlaboratory variation had little impact on our findings.

Concern about interlaboratory variability in Ki67 scores has limited the adoption of Ki67 into treatment guidelines and clinical practice (1,9,10). We have demonstrated that standardizing Ki67 scores for interlaboratory variation made little difference to our results. This finding suggests that, in practice, interlaboratory variation may be less of a problem than sometimes thought. In addition, initiatives now underway to improve Ki67 scoring methods in breast cancer may further reduce the effect of interlaboratory variation (1,11,12).

Although many studies have identified the prognostic properties of Ki67 in early breast cancer, these studies vary substantially regarding the selection of patients, the tumor molecular subtypes included, and the oncological outcomes considered (Supplementary Figure 13, Supplementary Table 5, available online). Many early studies also used a single cut point to demarcate women with “high-risk” cancers, and the cut points chosen varied across studies. These issues make pooled analyses of the literature challenging (4).

The decision to recommend chemotherapy in ER-positive, HER2-negative disease either before or after surgery is often difficult. As a result, several commercial multigene assays have been developed and are widely used to estimate the risk of distant recurrence (13-17). Many of these are expensive and require central analysis of tissue with accompanying time delays, and they may not be accessible to all centers (18). Ki67, combined with other routinely reported molecular markers (ER, PR, and HER2), has been proposed as a more accessible, less expensive, and equally informative alternative (19). Our study provides real-world evidence in support of the use of Ki67 in this role.

The IKWG consensus recently recommended that multigene assays to determine prognosis for patients with ER-positive breast cancer were not needed for those with low (≤5%) or very high (≥30%) Ki67 scores, but that interlaboratory concordance was insufficient for intermediate values to be considered reliable (2). Our findings confirm the increase in breast cancer mortality rate with increasing Ki67. However, we have also revealed that there is considerable digit preference in Ki67 scores (Figure 1), suggesting that patients would be more effectively separated into groups with different risks if the cut points were set at values that are not subject to digit preference (eg, 7/8, 17/18, 27/28, etc).

Other roles of Ki67 in breast cancer management have also emerged in recent years, such as triaging care during the Covid-19 pandemic, where Ki67 has been proposed as an objective method of identifying women who should have a high priority for surgery or neoadjuvant chemotherapy when access to these treatments is limited (20,21). Our findings confirm the prognostic value of Ki67 in such situations, even where information on extent of nodal involvement and pathological tumor size are not available.

This study is to our knowledge the largest to date to characterize the risks of breast cancer mortality according to Ki67 score and the first to examine its prognostic value in routine care in more than 20 centers without the use of a central laboratory for determination of Ki67 scores. Our analyses were confined to women who received surgery for early breast cancer and are therefore not directly generalizable to women with metastatic disease.

The sensitivity of methods to detect Ki67 has increased steadily over time. However, because we adjusted for calendar year in our analyses, the increase should have little effect on our results. We have considered breast cancer mortality only and not IBC recurrences because reliable population-based information on breast cancer recurrences in England is lacking. Other limitations in our data were missing values for some patient and tumor characteristics, but apart from PR status, this affected only a small proportion of women. Data on Ki67 scores throughout England have been recorded within NDRS only in recent years, and therefore follow-up information for these women is limited to 8 years, as reported in this article. In the future, further follow-up will allow evaluation of how these trends develop in the longer term.

Of the cut points widely cited in the literature, we have shown that Ki67 values of 30% or more in particular, are strongly associated with higher breast cancer mortality. One of the main factors limiting the routine use of Ki67 in breast cancer is concern over interlaboratory variation. Our study has confirmed that although this variation is certainly present, Ki67 nevertheless has strong prognostic value for breast cancer mortality in women with ER-positive, HER2-negative early IBC in the general hospital setting, even after adjusting for a wide range of patient and tumor factors. Our results may help to bring Ki67 into wider use as a factor considered in routine decision making in many aspects of breast cancer treatment. Coordinated international work is currently ongoing to reach agreement and improve standardized assessment and reporting of Ki67. Numerical methods of standardization, such as the one we have used, could complement this work. This should refine the prognostic value of Ki67 and its clinical utility. More work is also required to support the routine reporting of Ki67, as well as validating its potential in comparison with commonly used gene expression molecular profiling assays.

## Supplementary Material

pkad054_Supplementary_Data

## Data Availability

Depersonalized study data may be made available on request to accredited researchers who submit a proposal that is approved by the National Disease Registration Service Office for Data Release.
