# Supplementary material for: Ki67 and breast cancer mortality in women with invasive breast cancer
Source: JNCI Cancer Spectr. 2023 Aug 11;7(5):pkad054. doi: 10.1093/jncics/pkad054 (PMC10500622; doi:10.1093/jncics/pkad054)
Supplement: pkad054_Supplementary_Data [file pkad054_supplementary_data.pdf]

Ki67 and Breast Cancer Mortality in Women with Invasive Breast Cancer

Supplementary material

Supplementary Methods

Supplementary Methods	Statistical Methods and Results

Supplementary Tables

Supplementary Table 1	Distribution of Ki67 samples by source of Ki67 score
Supplementary Table 2	Distribution of women diagnosed with early breast cancer who had a Ki67 score recorded at diagnosis, by ER and HER2 status and patient and tumor characteristics
Supplementary Table 3	Cumulative breast cancer mortality risks in women with ER-positive and HER2-negative breast cancer by time since diagnosis and percentage Ki67 score
Supplementary Table 4	Breast cancer mortality rate ratios by Ki67 score in women with ER-positive and HER2-negative breast cancer showing effect of adjustment for each variable
Supplementary Table 5	Cohort studies reporting the association with Ki67 score and outcomes in women with early breast cancer

Supplementary Figures

Supplementary Figure 1	Derivation of study population
Supplementary Figure 2	Quantile-quantile plots of the crude and log-transformed Ki67 scores for each of the 25 pathology laboratories
Supplementary Figure 3	Distribution of Ki67 scores among the study population, for all women and by ER and HER2 status
Supplementary Figure 4	Crude breast cancer mortality rates and rate ratios by Ki67 score, by ER and HER2 status
Supplementary Figure 5	Crude all-cause mortality rates and rate ratios by Ki67 score, by ER and HER2 status
Supplementary Figure 6	Breast cancer mortality rate ratios for women diagnosed with ER-positive and HER2-negative disease with all available characteristics
Supplementary Figure 7	Breast cancer mortality rate ratios for women diagnosed with ER-positive and HER2-negative disease with characteristics that would be available when considering patients for neoadjuvant therapy
Supplementary Figure 8	All-cause mortality rate ratios by Ki67 score in ER-positive and HER2-negative breast cancer, and comparison using current clinical guidelines
Supplementary Figure 9	Cumulative all-cause mortality risks by Ki67 score, calculated from crude rates, rates adjusted for all variables shown in Table 1 and rates adjusted for all variables except tumor size and number of positive node
Supplementary Figure 10	Laboratory-standardized breast cancer mortality rate ratios for women diagnosed with ER-positive and HER2-negative early breast cancer with all available characteristics
Supplementary Figure 11	Laboratory-standardized breast cancer mortality rate ratios for women diagnosed with ER-positive and HER2-negative early breast cancer with characteristics that would be available when considering patients for neoadjuvant therapy
Supplementary Figure 12	Sensitivity analysis: Breast cancer mortality rate ratios by Ki67 score in women with and without a record of receiving chemotherapy treatment by Ki67 score classified into six groups avoiding the use of preferred digits as cut-points
Supplementary Figure 13	The process of study identification for the literature review

Supplementary Methods

Supplementary Methods: Statistical Methods

A. Tabulation of person-years at risk & observed events

For each woman who was eligible for the study, the length of time from 3 months after her cancer diagnosis until the earliest of death, emigration, or 31st December 2020 was calculated. These lengths of time were added together to form the person-years at risk. The number of women whose contribution to the person-years was terminated by death from breast cancer was also obtained, while women whose contribution to the person-years was terminated by emigration or the end of follow-up were censored, as were women who died from a cause other than breast cancer (apart from the analyses that considered deaths from all causes). The numbers of person-years were then tabulated simultaneously according to all the factors shown in Table 1, as were the numbers of deaths.

B. Annual breast cancer mortality rates and rate ratios

Crude annual breast cancer mortality rates were calculated by dividing the number of deaths observed in a particular group by the number of person-years at risk, and their associated confidence intervals were calculated by assuming that the number of deaths observed had a Poisson distribution. Crude rate ratios were estimated using Poisson regression with the numbers of deaths as the dependent variable, the numbers of person-years (which were assumed to be fixed) as the exposure, and the variable of interest (e.g. Ki67) included as a categorical factor. Time since diagnosis was also included as a categorical factor by classifying both the person-years and the numbers of deaths into the following categories: 0.25-, 1-, 2-, ...7-, 8+ years. Adjusted rate ratios were estimated by including these factors and also all the other characteristics listed in Table 1 in the model simultaneously, using the categories displayed in Table 1. For each factor, missing values were assigned to a separate category. Significance tests were carried out using the likelihood ratio omitting the categories for missing values. Group-specific 95% confidence intervals were calculated for the rate ratios for each category of each factor (Plummer M. Improved estimates of floating absolute risk. *Stat. Med.* 2004; 23:93-104).

To obtain adjusted annual mortality rates (rather than rate ratios), a Poisson regression model with main effects for Ki67 and all the factors to be included in the adjustment was fitted. Then, for each category of a particular characteristic, a weighted average of the estimates involving that category was calculated, with weights proportional to the person-years.

C. Adjusted cumulative risk of breast cancer mortality

If $\widehat{\lambda_i}$ denotes the estimated adjusted annual breast cancer mortality rate for time-interval i (as calculated in section B above), and $\hat{\sigma}_i^2$ denotes the variance of $\widehat{\lambda_i}$, where w_i is the length of the ith time interval, then the cumulative rate, and its corresponding variance, were calculated as

$$\widehat{\Lambda} = \sum_{j=1}^{J} w_j \widehat{\lambda_j} , \qquad \mathrm{var}(\widehat{\Lambda}) = \sum_{j=1}^{J} {w_j}^2 \hat{\sigma}_j^2 .$$

The cumulative rates (point estimates and 95% confidence limits) were then transformed into the cumulative risk using the formula, $\hat{P} = 1 - \exp(-\widehat{\Lambda})$.

D. Standardization between laboratories

For the women with ER-positive and HER2-negative disease, an investigation of the distribution of Ki67 measurements from each laboratory was conducted to assess whether there was any evidence of systematic differences between them. A box and whisker plot showed that the median of the Ki67 measurements varied between laboratories by a factor of more than 4 (from 5% to 22%) (Figure 5A), and a Kruskal-Wallis rank test (allowing for ties) also rejected the hypothesis that the distribution of the Ki67 measurements was identical in the different laboratories (p<0.001).

The distributions of Ki67 measurements within each laboratory were also highly skewed with a heavy upper tail. Therefore, before adjusting for the difference in median values between laboratories, it was desirable to find a transformation that removed the skewness. This was done with the aid of quantile-quantile plots. In these plots the Ki67 scores in each laboratory were sorted into rank order ($x_{(1)}$, $x_{(2)}$, $x_{(3)}$, ..., $x_{(N)}$), where N is the number of observations in the laboratory, and the ranked values were plotted against a scaled inverse normal distribution. To obtain the appropriate scaling for each laboratory, the mean (μ) and variance (σ^2) of the Ki67 scores in the laboratory were calculated. Then, if $\Phi_{(i)}$ is the inverse of the standard normal distribution corresponding to $x_{(i)}$ (i.e. (i/N+1)), the scaled value of $x_{(i}$ was

$$\mu + \sigma^2\Phi_{(i)}.$$

The quantile-quantile plots for the raw Ki67 scores are shown in Supplementary Figure 2A. A number of different transformations were investigated and it was found that transforming the Ki67 measurements by taking natural logarithms (plus one to avoid a singularity with measurements of zero) resulted in distributions that were approximately symmetric (Figure 5B) and also approximately normally distributed, see Supplementary Figure 2B.

After transformation, a one-way analysis of variance showed that there were still highly significant differences between the means of the transformed measurements from the laboratories ($F(24,8187)=49.66$, p<0.0001). These differences were removed by subtracting from each transformed measurement the mean value of the laboratory-specific transformed measurements, and then adding to it the mean value over all the transformed measurements. In these standardized values, the laboratory-specific median values varied by only a factor of 1.2 and there was little evidence of skewness (Figure 5C). In addition, a Kruskal-Wallis rank test (allowing for ties) provided no evidence to reject the hypothesis that the distributions of the transformed measurements were identical in the different laboratories (p=0.96). The standardized scores were back-transformed to the percentage scale by exponentiating and subtracting one (3 women had scores below 0 and 33 above 100). These back-transformed scores were rounded to the nearest integer value before being grouped using the same cut-points as in the unstandardized analyses. The upper bounds of the highest Ki67 groups was left unrestricted, because 31 women had rounded standardized scores above 100.

E. Digit preference in Ki67 recording

The recorded Ki67 scores exhibited considerable digit preference, with the majority of scores reported as 5, 10, 15, etc. (Figure 1). This suggests that, for example, many women for whom a score of 5 is reported, may actually have a score of 3, 4, 6, or 7. Therefore, to provide a potentially more robust and separation of the women into groups with different risks, grouped the Ki67 scores into categories that did not use the preferred digits as cut-points: 0-7%, 8-17%, 18-27%, 28-37%, 38-57%, and 58-100%. Sensitivity analyses were carried out to establish if the digit preference has altered our results.

Supplementary Methods: Additional Results

F. Molecular subtypes and breast cancer mortality

For all women included in the study, 8212 (62.4%) had ER-positive, HER2-negative disease, whilst 877 (6.7%) had ER-positive, HER2-positive disease, 986 (7.5%) had ER-negative, HER2-negative disease and 323 (2.4%) had ER-negative, HER2-positive disease. (Supplementary Table 2 and Supplementary Figure 3, panels B-E). A further 2761 (21.0%) women had either HER2-status or ER-status unknown (Supplementary Figure 3, panel F). Further characteristics of the study population are shown in Supplementary Table 2.

During a median follow-up of 6.2 years, 1516 women died, including 621 breast cancer deaths. For women with ER-positive, HER2-negative disease, the crude breast cancer mortality rate increased with Ki67 score (p_{trend}<0.001). For women with ER-positive, HER2-positive or ER-negative, HER2-positive disease there was no significant association between Ki67 score and crude breast cancer mortality rate. While for women with ER-negative, HER2-negative disease (p_{trend}=0.01) there was a significant association (Supplementary Figure 4), although the deviating group, [0-5], had fewer than 6% (58/986) of the women in. When the analysis was repeated considering deaths from all causes, the results were similar for but the association seen in ER-negative, HER2-negative disease was no longer present (p_{trend}=0.22) (Supplementary Figure 5). Among women with unknown ER or HER2, both the distribution of Ki67 scores and the trends in breast cancer mortality rate and all-cause mortality rate with Ki67 score were similar to those for women with ER-positive, HER2-negative disease, reflecting the fact that this group is the commonest of the four molecular subtypes (Supplementary Figure 3 and Supplementary Table 2).

Supplementary Tables

Supplementary Table 1i. Distribution of Ki67 samples by source of Ki67 score, according to ER and HER2 status and calendar period of diagnosis. Ki67 sample date linked to nearest pathology investigation type within ±1 week.

Characteristics	No. of women (%) by source of Ki67			Total no. of women
	Biopsy	Surgical specimen	Missing	
ER & HER2 status				
ER-positive & HER2-negative	2910 (35%)	896 (11%)	4406 (54%)	8212
ER-positive & HER2-positive	254 (29%)	77 (9%)	546 (62%)	877
ER-negative & HER2-negative	326 (33%)	101 (10%)	559 (57%)	986
ER-negative & HER2-positive	108 (33%)	22 (7%)	193 (60%)	323
ER or HER2 unknown	757 (28%)	423 (15%)	1581 (57%)	2761
Calendar period of diagnosis				
2009-2012	46 (2%)	46 (2%)	3064 (97%)	3154
2013-2014	345 (7%)	165 (4%)	4174 (89%)	4684
2015-2016	3966 (75%)	1308 (24%)	47 (1%)	5321
**Total no. of women (%)**	**4355 (33%)**	**1519 (12%)**	**7285 (55%)**	**13,159**

Supplementary Table 1ii. Distribution of Ki67 samples by source of Ki67 score, according to calendar period of diagnosis and age at diagnosis, in women with ER-positive and HER2-negative breast cancer. Ki67 sample date linked to nearest pathology investigation type within ±1 week.

Characteristics	No. of women (%) with ER-positive and HER2-negative disease by source of Ki67			Total no. of women
	Biopsy	Surgical specimen	Missing	
Calendar period of diagnosis				
2009-2012	6 (<1%)	9 (<1%)	1459 (99%)	1474
2013-2014	259 (8%)	118 (4%)	2915 (88%)	3292
2015-2016	2645 (77%)	769 (22%)	32 (1%)	3446
Age at diagnosis (years)				
18-39	79 (32%)	23 (9%)	149 (59%)	251
40-49	500 (36%)	139 (10%)	747 (54%)	1386
50-64	1205 (34%)	413 (12%)	1879 (54%)	3497
65-70	563 (36%)	153 (10%)	835 (54%)	1551
71-79	413 (37%)	124 (11%)	570 (52%)	1107
80-89	150 (36%)	44 (10%)	226 (54%)	420
**Total no. of women (%)**	**2910 (35%)**	**869 (11%)**	**4406 (54%)**	**8212**

Supplementary Table 2. Distribution of women with early breast cancer who had a Ki67 score recorded at or shortly after diagnosis, by ER and HER2 status and patient and tumor characteristics.

Characteristic	Column percent of women (%)					Total %	Total number of women
	ER-positive		ER-negative		ER or HER2 unknown		
	HER2-negative	HER2-positive	HER2-negative	HER2-positive			
**Calendar period of diagnosis** (p<0.001)							
2009–2012	18	25	21	23	43	24	3154
2013–2014	40	41	40	42	18	36	4684
2015–2016	42	34	39	35	39	40	5321
**Age at diagnosis (years)** (p<0.001)							
18–39	3	7	7	7	5	4	546
40–49	17	20	17	15	19	17	2298
50–64	43	43	36	35	38	41	5396
65–70	19	16	16	14	17	18	2376
71–79	13	10	15	20	14	14	1786
80–89	5	4	9	9	7	6	757
**Cancer screen-detected** (p<0.001)							
Screen-detected	40	32	22	25	31	36	4735
Not screen-detected	60	68	78	75	69	64	8424
**Tumor size (mm)** (p<0.001)							
1–20	59	52	44	44	50	55	7273
21–50	29	37	43	40	30	31	4101
>50	4	3	4	6	3	4	495
Unknown	8	8	9	10	17	10	1290
**Number of positive nodes** (p<0.001)							
0	65	61	63	55	62	65	8458
1 to 3	23	25	21	24	20	22	2936
4 to 9	4	5	4	7	5	4	578
10 or more	2	3	3	5	3	2	288
Unknown	6	6	9	9	10	7	899
**Tumor grade** (p<0.001)							
Low	20	5	2	1	15	16	2149
Medium	62	46	18	19	51	54	7144
High	18	48	79	79	32	29	3765
Unknown	0	1	1	1	2	1	101
**PR-status** (p<0.001)							
Positive	68	59	9	7	16	51	6656
Negative	9	20	84	87	6	16	2168
Unknown	23	21	7	6	78	33	4335
**Total %**	100	100	100	100	100	100	—
**Total number of women**	8212	877	986	323	2761	—	13,159
**Ki67 median (IQR)**	12 (5,23)	25 (14,40)	50 (26,71)	36 (24,55)	16 (9,31)	—	15 (7,30)
**Ki67 mean (min,max)**	17 (0,100)	29 (0,90)	49 (1,100)	40 (1,95)	24 (0,100)	—	22 (0,100)

Abbreviations: PR progesterone receptor; IQR interquartile range. P-values are for heterogeneity.

Supplementary Table 3i. Cumulative breast cancer mortality risks in women with ER-positive and HER2-negative breast cancer by time since diagnosis and percentage Ki67 score. Estimates given for crude risks, adjusted risks, and adjusted risks excluding tumor size and number of positive nodes.

				Crude risks		Adjusted risks		Adjusted risks (excluding tumor size & positive nodes)	
Ki67 category	Time since diagnosis	Number of women at risk	Number of breast cancer deaths since previous time-point	Cumulative mortality % (95% CI)	Absolute increase in cumulative mortality vs. lowest Ki67 category	Cumulative mortality % (95% CI)	Absolute increase in cumulative mortality vs. lowest Ki67 category	Cumulative mortality % (95% CI)	Absolute increase in cumulative mortality vs. lowest Ki67 category
0-5	3 months	2258	—	0.0 (0.0 to 0.0)	[Reference]	0.0 (0.0 to 0.0)	[Reference]	0.0 (0.0 to 0.0)	[Reference]
0-5	1 year	2248	5	0.1 (0.1 to 0.2)	[Reference]	0.1 (0.1 to 0.2)	[Reference]	0.1 (0.1 to 0.2)	[Reference]
0-5	2 years	2235	3	0.3 (0.2 to 0.4)	[Reference]	0.4 (0.3 to 0.6)	[Reference]	0.4 (0.3 to 0.6)	[Reference]
0-5	3 years	2219	2	0.6 (0.5 to 0.8)	[Reference]	0.8 (0.6 to 1.1)	[Reference]	0.8 (0.6 to 1.0)	[Reference]
0-5	4 years	2195	5	0.9 (0.7 to 1.1)	[Reference]	1.2 (1.0 to 1.5)	[Reference]	1.1 (0.9 to 1.4)	[Reference]
0-5	5 years	1786	6	1.2 (0.9 to 1.4)	[Reference]	1.7 (1.4 to 2.1)	[Reference]	1.6 (1.3 to 1.9)	[Reference]
0-5	6 years	1277	8	1.5 (1.2 to 1.8)	[Reference]	2.2 (1.8 to 2.7)	[Reference]	2.0 (1.7 to 2.5)	[Reference]
0-5	7 years	793	4	1.9 (1.6 to 2.2)	[Reference]	2.9 (2.4 to 3.5)	[Reference]	2.7 (2.2 to 3.2)	[Reference]
0-5	8 years	409	1	2.1 (1.8 to 2.5)	[Reference]	3.3 (2.8 to 4.0)	[Reference]	3.0 (2.5 to 3.7)	[Reference]
6-10	3 months	1723	—	0.0 (0.0 to 0.0)	0.00	0.0 (0.0 to 0.0)	0.00	0.0 (0.0 to 0.0)	0.00
6-10	1 year	1717	1	0.1 (0.1 to 0.2)	0.03	0.1 (0.1 to 0.3)	0.01	0.1 (0.1 to 0.2)	0.01
6-10	2 years	1704	4	0.4 (0.3 to 0.6)	0.09	0.5 (0.3 to 0.7)	0.04	0.5 (0.3 to 0.7)	0.04
6-10	3 years	1687	4	0.8 (0.6 to 1.0)	0.18	0.9 (0.7 to 1.2)	0.08	0.9 (0.7 to 1.1)	0.08
6-10	4 years	1660	6	1.1 (0.9 to 1.4)	0.25	1.3 (1.0 to 1.7)	0.12	1.3 (1.0 to 1.6)	0.12
6-10	5 years	1313	7	1.5 (1.2 to 1.8)	0.34	1.8 (1.5 to 2.3)	0.17	1.7 (1.4 to 2.1)	0.16
6-10	6 years	945	5	1.9 (1.6 to 2.3)	0.44	2.4 (2.0 to 2.9)	0.22	2.3 (1.9 to 2.7)	0.21
6-10	7 years	575	6	2.4 (2.0 to 2.9)	0.55	3.2 (2.6 to 3.8)	0.28	2.9 (2.4 to 3.5)	0.27
6-10	8 years	280	1	2.8 (2.3 to 3.3)	0.63	3.7 (3.0 to 4.4)	0.33	3.3 (2.8 to 4.0)	0.31
11-19	3 months	1484	—	0.0 (0.0 to 0.0)	0.00	0.0 (0.0 to 0.0)	0.00	0.0 (0.0 to 0.0)	0.00
11-19	1 year	1478	3	0.1 (0.1 to 0.2)	0.03	0.1 (0.1 to 0.2)	0.00	0.1 (0.1 to 0.2)	0.01
11-19	2 years	1468	4	0.4 (0.3 to 0.6)	0.09	0.4 (0.3 to 0.6)	0.01	0.4 (0.3 to 0.6)	0.03
11-19	3 years	1449	6	0.8 (0.6 to 1.0)	0.17	0.8 (0.6 to 1.1)	0.02	0.8 (0.6 to 1.1)	0.05
11-19	4 years	1428	4	1.1 (0.9 to 1.4)	0.25	1.2 (1.0 to 1.6)	0.02	1.2 (1.0 to 1.6)	0.08
11-19	5 years	1121	6	1.5 (1.2 to 1.9)	0.34	1.7 (1.4 to 2.1)	0.03	1.7 (1.4 to 2.1)	0.11
11-19	6 years	755	5	1.9 (1.6 to 2.3)	0.43	2.2 (1.8 to 2.7)	0.04	2.2 (1.8 to 2.7)	0.14
11-19	7 years	433	2	2.4 (2.0 to 2.9)	0.54	2.9 (2.4 to 3.6)	0.05	2.8 (2.3 to 3.4)	0.18
11-19	8 years	189	0	2.8 (2.3 to 3.3)	0.62	3.4 (2.8 to 4.1)	0.06	3.2 (2.7 to 3.9)	0.20

Supplementary Table 3i continued.

Ki67 category	Time since diagnosis	Number of women at risk	Number of breast cancer deaths since previous time-point	Crude risks		Adjusted risks		Adjusted risks (excluding tumor size & positive nodes)	
				Cumulative mortality % (95% CI)	Absolute increase in cumulative mortality vs. lowest Ki67 category	Cumulative mortality % (95% CI)	Absolute increase in cumulative mortality vs. lowest Ki67 category	Cumulative mortality % (95% CI)	Absolute increase in cumulative mortality vs. lowest Ki67 category
20-29	3 months	1304	—	0.0 (0.0 to 0.0)	0.00	0.0 (0.0 to 0.0)	0.00	0.0 (0.0 to 0.0)	0.00
20-29	1 year	1296	3	0.2 (0.1 to 0.3)	0.07	0.1 (0.1 to 0.2)	0.00	0.1 (0.1 to 0.2)	0.01
20-29	2 years	1284	4	0.5 (0.4 to 0.8)	0.24	0.4 (0.3 to 0.6)	0.01	0.5 (0.3 to 0.7)	0.05
20-29	3 years	1264	5	1.0 (0.8 to 1.4)	0.45	0.8 (0.6 to 1.1)	0.03	0.9 (0.7 to 1.2)	0.09
20-29	4 years	1232	9	1.5 (1.2 to 1.9)	0.64	1.2 (1.0 to 1.6)	0.04	1.3 (1.0 to 1.6)	0.13
20-29	5 years	983	4	2.0 (1.7 to 2.5)	0.87	1.7 (1.4 to 2.1)	0.05	1.8 (1.4 to 2.1)	0.18
20-29	6 years	671	3	2.6 (2.1 to 3.1)	1.11	2.3 (1.9 to 2.7)	0.07	2.3 (1.9 to 2.8)	0.24
20-29	7 years	403	4	3.3 (2.7 to 3.9)	1.40	3.0 (2.5 to 3.6)	0.09	3.0 (2.5 to 3.6)	0.31
20-29	8 years	191	3	3.7 (3.1 to 4.4)	1.59	3.4 (2.8 to 4.1)	0.10	3.4 (2.8 to 4.1)	0.35
30-39	3 months	652	—	0.0 (0.0 to 0.0)	0.00	0.0 (0.0 to 0.0)	0.00	0.0 (0.0 to 0.0)	0.00
30-39	1 year	648	0	0.3 (0.2 to 0.6)	0.24	0.2 (0.1 to 0.4)	0.07	0.2 (0.1 to 0.4)	0.10
30-39	2 years	642	3	1.1 (0.8 to 1.6)	0.80	0.7 (0.5 to 1.0)	0.24	0.7 (0.5 to 1.1)	0.33
30-39	3 years	627	10	2.1 (1.6 to 2.8)	1.51	1.3 (1.0 to 1.7)	0.45	1.4 (1.1 to 1.9)	0.63
30-39	4 years	614	5	3.0 (2.4 to 3.8)	2.16	1.9 (1.5 to 2.4)	0.66	2.1 (1.6 to 2.6)	0.91
30-39	5 years	486	7	4.1 (3.3 to 5.0)	2.92	2.6 (2.1 to 3.2)	0.91	2.8 (2.3 to 3.4)	1.24
30-39	6 years	320	6	5.2 (4.3 to 6.2)	3.70	3.4 (2.8 to 4.1)	1.20	3.6 (3.0 to 4.4)	1.61
30-39	7 years	184	4	6.5 (5.5 to 7.8)	4.66	4.5 (3.7 to 5.4)	1.57	4.7 (3.9 to 5.7)	2.08
30-39	8 years	87	1	7.4 (6.2 to 8.8)	5.28	5.1 (4.3 to 6.2)	1.80	5.4 (4.5 to 6.5)	2.36
40-100	3 months	791	—	0.0 (0.0 to 0.0)	0.00	0.0 (0.0 to 0.0)	0.00	0.0 (0.0 to 0.0)	0.00
40-100	1 year	780	4	0.7 (0.4 to 1.2)	0.60	0.3 (0.2 to 0.5)	0.18	0.4 (0.2 to 0.6)	0.23
40-100	2 years	760	18	2.3 (1.7 to 3.0)	1.95	1.0 (0.7 to 1.4)	0.59	1.2 (0.9 to 1.6)	0.77
40-100	3 years	735	19	4.3 (3.4 to 5.3)	3.67	1.9 (1.5 to 2.5)	1.13	2.2 (1.8 to 2.9)	1.46
40-100	4 years	713	13	6.1 (5.1 to 7.3)	5.24	2.8 (2.3 to 3.5)	1.64	3.2 (2.6 to 4.0)	2.10
40-100	5 years	571	14	8.2 (7.0 to 9.6)	7.03	3.9 (3.3 to 4.7)	2.26	4.4 (3.7 to 5.3)	2.86
40-100	6 years	396	7	10.3 (8.9 to 11.9)	8.85	5.2 (4.4 to 6.1)	2.96	5.7 (4.9 to 6.8)	3.70
40-100	7 years	231	8	12.9 (11.3 to 14.9)	11.07	6.7 (5.7 to 7.9)	3.85	7.4 (6.3 to 8.7)	4.77
40-100	8 years	107	4	14.6 (12.7 to 16.8)	12.49	7.7 (6.6 to 9.1)	4.41	8.5 (7.2 to 10.0)	5.42

Supplementary Table 3ii. Cumulative breast cancer mortality risks in women with ER-positive and HER2-negative breast cancer by time since diagnosis and percentage Ki67 score Estimates given for crude risks, adjusted risks, and adjusted risks excluding tumor size and number of positive nodes.

				Crude risks		Adjusted risks		Adjusted risks (excluding tumor size & positive nodes)	
Ki67 category	Time since diagnosis	Number of women at risk	Number of breast cancer deaths since previous time-point	Cumulative mortality % (95% CI)	Absolute increase in cumulative mortality vs. lowest Ki67 category	Cumulative mortality % (95% CI)	Absolute increase in cumulative mortality vs. lowest Ki67 category	Cumulative mortality % (95% CI)	Absolute increase in cumulative mortality vs. lowest Ki67 category
0-5	3 months	2258	—	0.0 (0.0 to 0.0)	[Reference]	0.0 (0.0 to 0.0)	[Reference]	0.0 (0.0 to 0.0)	[Reference]
0-5	1 year	2248	5	0.1 (0.1 to 0.2)	[Reference]	0.1 (0.1 to 0.2)	[Reference]	0.1 (0.1 to 0.2)	[Reference]
0-5	2 years	2235	3	0.3 (0.2 to 0.4)	[Reference]	0.4 (0.3 to 0.6)	[Reference]	0.4 (0.3 to 0.6)	[Reference]
0-5	3 years	2219	2	0.6 (0.5 to 0.8)	[Reference]	0.8 (0.6 to 1.1)	[Reference]	0.8 (0.6 to 1.1)	[Reference]
0-5	4 years	2195	5	0.9 (0.7 to 1.1)	[Reference]	1.2 (1.0 to 1.5)	[Reference]	1.2 (0.9 to 1.5)	[Reference]
0-5	5 years	1786	6	1.2 (0.9 to 1.4)	[Reference]	1.7 (1.4 to 2.1)	[Reference]	1.6 (1.3 to 2.0)	[Reference]
0-5	6 years	1277	8	1.5 (1.2 to 1.8)	[Reference]	2.2 (1.8 to 2.7)	[Reference]	2.1 (1.7 to 2.5)	[Reference]
0-5	7 years	793	4	1.9 (1.6 to 2.2)	[Reference]	2.9 (2.4 to 3.5)	[Reference]	2.7 (2.3 to 3.3)	[Reference]
0-5	8 years	409	1	2.1 (1.8 to 2.5)	[Reference]	3.4 (2.8 to 4.1)	[Reference]	3.1 (2.6 to 3.7)	[Reference]
6-29	3 months	4511	—	0.0 (0.0 to 0.0)	0.00	0.0 (0.0 to 0.0)	0.00	0.0 (0.0 to 0.0)	0.00
6-29	1 year	4491	7	0.1 (0.1 to 0.2)	0.04	0.1 (0.1 to 0.2)	0.01	0.1 (0.1 to 0.2)	0.01
6-29	2 years	4456	12	0.4 (0.3 to 0.6)	0.13	0.5 (0.3 to 0.6)	0.02	0.5 (0.3 to 0.6)	0.04
6-29	3 years	4400	15	0.8 (0.7 to 1.1)	0.25	0.9 (0.7 to 1.1)	0.04	0.9 (0.7 to 1.1)	0.07
6-29	4 years	4320	19	1.2 (1.0 to 1.5)	0.36	1.3 (1.1 to 1.5)	0.06	1.3 (1.1 to 1.5)	0.10
6-29	5 years	3417	17	1.7 (1.4 to 1.9)	0.49	1.8 (1.5 to 2.1)	0.08	1.7 (1.5 to 2.0)	0.14
6-29	6 years	2371	13	2.1 (1.8 to 2.4)	0.63	2.3 (2.0 to 2.7)	0.10	2.3 (1.9 to 2.6)	0.18
6-29	7 years	1411	12	2.7 (2.3 to 3.1)	0.79	3.1 (2.6 to 3.6)	0.13	2.9 (2.5 to 3.4)	0.23
6-29	8 years	660	4	3.0 (2.6 to 3.5)	0.90	3.5 (3.0 to 4.1)	0.15	3.4 (2.9 to 3.9)	0.26
30-100	3 months	1443	—	0.0 (0.0 to 0.0)	0.00	0.0 (0.0 to 0.0)	0.00	0.0 (0.0 to 0.0)	0.00
30-100	1 year	1428	4	0.5 (0.3 to 0.9)	0.44	0.3 (0.2 to 0.4)	0.13	0.3 (0.2 to 0.5)	0.17
30-100	2 years	1402	21	1.7 (1.3 to 2.3)	1.43	0.9 (0.6 to 1.2)	0.42	1.0 (0.7 to 1.3)	0.55
30-100	3 years	1362	29	3.3 (2.7 to 4.0)	2.69	1.7 (1.3 to 2.1)	0.81	1.9 (1.5 to 2.3)	1.06
30-100	4 years	1327	18	4.7 (3.9 to 5.6)	3.85	2.4 (2.0 to 2.9)	1.19	2.7 (2.2 to 3.2)	1.52
30-100	5 years	1057	21	6.3 (5.4 to 7.4)	5.17	3.3 (2.8 to 3.9)	1.64	3.7 (3.1 to 4.3)	2.08
30-100	6 years	716	13	8.0 (7.0 to 9.2)	6.53	4.4 (3.8 to 5.1)	2.15	4.8 (4.1 to 5.6)	2.69
30-100	7 years	415	12	10.1 (8.8 to 11.5)	8.20	5.7 (4.9 to 6.7)	2.80	6.2 (5.3 to 7.2)	3.47
30-100	8 years	194	5	11.4 (9.9 to 13.1)	9.27	6.6 (5.6 to 7.7)	3.20	7.0 (6.0 to 8.2)	3.94

Supplementary Table 3iii. Cumulative breast cancer mortality risks in women with ER-positive and HER2-negative breast cancer by time since diagnosis and percentage Ki67 score Estimates given for crude risks, adjusted risks, and adjusted risks excluding tumor size and number of positive nodes.

				Crude risks		Adjusted risks		Adjusted risks (excluding tumor size & positive nodes)	
Ki67 category	Time since diagnosis	Number of women at risk	Number of breast cancer deaths since previous time-point	Cumulative mortality % (95% CI)	Absolute increase in cumulative mortality vs. lowest Ki67 category	Cumulative mortality % (95% CI)	Absolute increase in cumulative mortality vs. lowest Ki67 category	Cumulative mortality % (95% CI)	Absolute increase in cumulative mortality vs. lowest Ki67 category
0-10	3 months	3981	—	0.0 (0.0 to 0.0)	[Reference]	0.0 (0.0 to 0.0)	[Reference]	0.0 (0.0 to 0.0)	[Reference]
0-10	1 year	3965	6	0.1 (0.1 to 0.2)	[Reference]	0.1 (0.1 to 0.3)	[Reference]	0.1 (0.1 to 0.2)	[Reference]
0-10	2 years	3939	7	0.4 (0.3 to 0.5)	[Reference]	0.5 (0.4 to 0.7)	[Reference]	0.5 (0.3 to 0.7)	[Reference]
0-10	3 years	3906	6	0.7 (0.5 to 0.8)	[Reference]	0.9 (0.7 to 1.2)	[Reference]	0.9 (0.7 to 1.2)	[Reference]
0-10	4 years	3855	11	1.0 (0.8 to 1.2)	[Reference]	1.4 (1.1 to 1.7)	[Reference]	1.3 (1.1 to 1.6)	[Reference]
0-10	5 years	3099	13	1.3 (1.1 to 1.5)	[Reference]	1.9 (1.6 to 2.3)	[Reference]	1.8 (1.5 to 2.2)	[Reference]
0-10	6 years	2222	13	1.7 (1.4 to 1.9)	[Reference]	2.5 (2.1 to 3.0)	[Reference]	2.4 (2.0 to 2.8)	[Reference]
0-10	7 years	1368	10	2.1 (1.8 to 2.5)	[Reference]	3.3 (2.8 to 3.9)	[Reference]	3.1 (2.6 to 3.6)	[Reference]
0-10	8 years	689	2	2.4 (2.1 to 2.8)	[Reference]	3.8 (3.2 to 4.5)	[Reference]	3.5 (3.0 to 4.1)	[Reference]
11-100	3 months	4231	—	0.0 (0.0 to 0.0)	0.00	0.0 (0.0 to 0.0)	0.00	0.0 (0.0 to 0.0)	0.00
11-100	1 year	4202	10	0.3 (0.2 to 0.5)	0.17	0.2 (0.1 to 0.3)	0.04	0.2 (0.1 to 0.3)	0.06
11-100	2 years	4154	29	0.9 (0.7 to 1.2)	0.55	0.6 (0.5 to 0.8)	0.14	0.7 (0.5 to 0.9)	0.19
11-100	3 years	4075	40	1.7 (1.4 to 2.1)	1.04	1.2 (1.0 to 1.5)	0.26	1.3 (1.0 to 1.6)	0.36
11-100	4 years	3987	31	2.4 (2.1 to 2.9)	1.49	1.8 (1.5 to 2.1)	0.38	1.8 (1.6 to 2.2)	0.52
11-100	5 years	3161	31	3.3 (2.9 to 3.8)	2.01	2.4 (2.1 to 2.8)	0.53	2.5 (2.2 to 2.9)	0.71
11-100	6 years	2142	21	4.2 (3.7 to 4.8)	2.54	3.2 (2.8 to 3.7)	0.69	3.3 (2.9 to 3.8)	0.92
11-100	7 years	1251	18	5.3 (4.7 to 6.1)	3.21	4.2 (3.6 to 4.8)	0.90	4.3 (3.7 to 4.9)	1.19
11-100	8 years	574	8	6.0 (5.3 to 6.9)	3.64	4.8 (4.1 to 5.6)	1.03	4.9 (4.2 to 5.6)	1.36

Supplementary Table 4. Breast cancer mortality rate ratios by Ki67 score (%) in women with ER-positive and HER2-negative breast cancer showing effect of adjustment for each variable separately (i.e. set of models with univariate adjustments).

Variables included in adjustment	Rate ratios by Ki67 score					
	[0-5]	[6-10]	[11-19]	[20-29]	[30-39]	[40-100]
**No adjustment variables**	1.00	1.29	1.28	1.75	3.54	7.26
**Adjustment for one variable at a time**						
Tumor grade	1.00	1.16	1.07	1.18	1.92	3.33
Tumor size	1.00	1.28	1.26	1.53	2.68	5.01
Number of positive nodes	1.00	1.27	1.17	1.48	2.62	5.02
Cancer screen-detected	1.00	1.22	1.23	1.53	2.91	5.65
PR–status	1.00	1.32	1.33	1.80	3.56	6.47
Age at diagnosis	1.00	1.24	1.23	1.64	3.32	6.93
Ethnicity	1.00	1.29	1.28	1.74	3.48	7.17
Index of deprivation	1.00	1.29	1.27	1.72	3.51	7.21
Calendar period of diagnosis	1.00	1.30	1.29	1.75	3.55	7.28
Years since diagnosis	1.00	1.30	1.29	1.76	3.57	7.33
**Adjustment for all variables simultaneously**	1.00	1.10	1.02	1.03	1.55	2.38

When adjustment is made for tumor grade only, the rate ratio is reduced by a larger amount than when adjustment is made for any other single variable. This shows that tumor grade is the strongest confounding variable.

Supplementary Table 5. Cohort studies reporting the association with Ki67 score and outcomes in women with early breast cancer

Study	No. of women with Ki67 score recorded	Median duration of follow–up (years)	Upper Ki67 cut off used	Outcome assessed	Rate ratio (95% CI)	Notes
Ali 2012	1599	9.2 [a]	1% [b]	BCSS	1.8 (1.3–2.5) [c]	[a] Mean duration of follow–up<br>[b] A cell was considered positive if there was any nuclear signal above background<br>[c] Hazard ratio from univariate analysis
Carbognin 2016	405	5.6	21%	DFS<br>OS	3.61 (1.35–9.63)<br>12.58 (4.12–38.23)	
Carbognin 2017	1097	14.6	4% & 14% [d]	DFS<br>OS	NS [d]<br>NS	[d] Ki67 cut–offs for predicting DFS were 4% for ILC & 14% for IDC. Hazard ratio not specified. Follow up was 14.6 years for DFS & 17.8 years for OS. DFS: 69.2% for ILC with Ki67 >4% vs 79.4% if Ki67 (<=4%) and 52.6.2% DFS for IDC with Ki67 >14% vs 84.0% if Ki67 (<=14%)
De Angelis 2017	1049	8.3	20%	DFS	2.23 (1.24 – 4)	
Engels 2013	822	10	1%	OS<br>RFP [e]	1.089 (0.816 – 1.453)<br>1.304 (0.815 – 2.087)	[e] Relapse–free period defined as the time from surgery until an event (locoregional recurrence and/or a distant recurrence, whichever came first)
Fallahzedah 2018	538	5	NS	OS	0.908 (0.3 – 2.75)	
Fasching 2019	3140	5	20%	DFS<br>OS	1.76 (1.44 – 2.15)<br>NS	
Haerslev 1996	487	>10 [f]	1%	OS	NS [g]	[f] Mean duration of follow–up.<br>[g] HR not specified but the number of independent prognostic significance of Ki67 noted. However, Ki67 had a RR 1.14 (SD 0.14) with RFP when stratified with nodal status.
Hafeez 2013	438	7.3	10%	IBRFS<br>OS<br>BCSM<br>DMFS	1.007 (0.464-2.186)<br>0.982 (0.537-1.798)<br>1.089 (0.543-2.186)<br>1.124 (0.608 – 2.078)	
Huang 2013	504 [h]	5.5	20%	OS<br>DFS	1.935 (1.018 – 3.677) [i]<br>1.693 (1.046 – 2.741) [i]	[h] Study examined women with triple negative breast cancer<br>[i] Hazard ratio from univariate analysis
Inwald 2013	3658	3.6	>45% [j]	DFS<br>OS	2.42 (1.87 – 3.15)<br>2.13 (1.58 – 2.88)	[j] Other cut–offs examined: 16, 26, 36%
Ishihara 2013	591	4.5	30%	CNSM	3.9 (1.3 – 12.9)	
Kurebayashi 2014	261	8.3	30%	BCSS	19.6 (1.4 – 250)	
Lee 2010	7578	3.9	20%	OS	1.654 (0.75 – 3.647) [k]	[k] HR in triple negative breast cancer was 0.485 (0.245 – 0.959).
Li 2014	450	3.8	>25%	MFS<br>OS	NS [l]<br>2.07 (1.08 – 3.95)	[l] HR for OS in entire cohort not specified, however for patients with 1–3 positive lymph nodes (n = 262) the HR for MFS was 3.27 (1.16-9.27) and for OS was 10.64 (1.16-9.27) on multivariate analysis.
Liikanen 2018	936 [m]	9.5	– [n]	DFS<br>OS	2.16 (1.54 – 3.04)<br>1.41 (1.11 – 1.79)	[m] Low risk group, i.e. unilateral pT1N0i breast cancer (with and without ITCs)<br>[n] Ki67 was treated as a continuous variable
Maisonneuve 2014	9415 [o]	8.1	20%	DFS	1.93 (1.45 – 2.58) [p]	[o] Study examined ER–positive, human epidermal growth factor receptor 2 (HER2)–negative early breast cancer and had undergone surgery at the European Institute of Oncology between 1994 and 2006.<br>[p] HR 1.93 (1.45 – 2.58) for pgR >= 20 and HR 1.96 (1.44 – 2.67 if pgR <20%).
Matsubara 2011	1166	5.1	10%	DFS<br>OS	1.707 (1.152 – 2.528)<br>1.830 (1.045 – 3.204)	

Supplementary Table 5 continued.

Study	No. of women with Ki67 score recorded	Median duration of follow–up (years)	Upper Ki67 cut off used	Outcome assessed	Rate ratio (95% CI)	Notes
Meattini 2014	1040	8.5	20%	DFS<br>OS	2.18 (1.28 – 3.73)<br>3.76 (1.73 – 8.14)	
Nakano 2015	813	4.3 [q]	50%	OS	1.877 (1.295 – 2.724)	[q] The median follow–up period started after recurrence, as the aim of this study was to determine the impact of subtype and the year of recurrence on the survival times of recurrent breast cancer.
Niikura 2014	971 [r]	3.5	>20%	RFS<br>OS	0.462 (0.276 – 0.774)<br>NS	[r] Of the 1331 primary breast cancer patients included in the study, 971 patients had oestrogen receptor (ER)–positive and HER2–negative tumors.
Pellikainen 2003	420	4.8	20%	BCSS<br>RFS	3.62 (2.05-6.41)<br>3.90 (2.20-6.93) [s]	[s] Ki67 >20 using combined HER2/AP-2 expression had a recurrence-free survival relative risk of 3.65 (1.72-7.21).
Rajan 2014	368	15.7	14%	DFS<br>BCSS	1.0 (0.98–1.03) [t]<br>1.01 (0.9–1.04) [t]	[t] Ki67 was treated as a continuous variable
Rasmy 2016	280	2.8	14% [u]	OS<br>DFS	– [u]	[u] Among the 4 centers in this study, 3 of them used 14% as the cut-off value, while 1 center used 25% as the cut-off value. HR was not explicitly stated however OS was 34.82 months (±0.30) for patients with low ki67 vs 32.41 months (±0..43) for high ki67 and DFS was 37.029 months (±0. 476) for patients with low vs 32.242 months (±0. 792) for high Ki67 scores significantly lower in patients with high Ki67 scores
Robertson 2018	299	10.3	20%	OS	1.748 (1.093–2.795) [v]	[v] HR when cases with Ki67 assessed using Immunohistochemistry in tumor resections were analysed. Cases with high Ki67 (≥20% cut–off) had a significantly higher hazard for all–cause mortality compared with cases with low Ki67 p=0.020. For cases assessed using ICC using the Ki67 (≥20% cut–off), the HR was not significant (HR 1.344, 95% CI 0.853 to 2.117, p=0.203
Rudolph 2003	273	8.3	25%	BCSS<br>MFS	3.98 (1.70–9.31)<br>2.20 (1.13–4.28)	
Shin 2018	289 [w]	6.3	14%	RFS<br>DMRS	5.95 (1.88– 18.79)<br>3.57 (1.02, 12.41)	[w] Unilateral ER–positive, HER2–negative, node–negative breast cancer larger than 5 mm.
Tan 2019	406	3.7	0.3%	OS<br>DFS	0.919 (0.648–1.303)<br>0.727 (0.549– 0.964)	
Tashima 2015	4329	6.8	20%	DFS<br>OS	1.014 (1.008–1.020) [x]<br>1.014 (1.008–1.020) [x]	[x] Ki67 was treated as a continuous variable
Thangarah 2017	802	3.3	20%	DFS<br>OS	2.85 (1.45–5.59)<br>NS	
Yang 2019	296	4.4	40%	DFS<br>OS	1.977 (1.179–3.314)<br>2.778 (1.339–5.763)	
Zong 2014	398 [y]	2.3	30%	DFS<br>OS	NS [y]<br>-	[y] Number of Luminal B/HER2– patients. Study mainly examining the association of patient and tumor characteristics with PR-status. High Ki–67 index was significantly associated with poorer 2–year DFS (98.0%Vs 92.4% Log–rank p= .013) among Luminal B/HER2– patients. However, no significant overall survival difference was detected between the high Ki–67 index group and the low Ki–67 index group among Luminal B/HER2– patients.
Zurrida 2013	285	11.1	14%	DFS<br>OS<br>CSH–LF<br>CSH–DM	1.41 (0.64–3.13)<br>1.34 (0.47–3.78)<br>1.54 (0.55–4.30)<br>1.19 (0.34–4.15)	

Supplementary Table 5 continued.

Study	No. of women with Ki67 score recorded	Median duration of follow–up (years)	Upper Ki67 cut off used	Outcome assessed	Rate ratio (95% CI)	Notes
Caleffi 2020	909	7	NS	OS	HR 5.46 (1.27-23.32)	HR relates to intermediate/high vs 'negative' Ki67 score although Ki67 cut-offs not specified.
Karakolevska-Ilova 2021	336	NS	>40%	OS	NS	Rate ratio not stated, but Ki-67 not significant (p>0.05)
Leonardi 2021	2362	8.2	20-49%<br>≥50%	BCSS	HR 1.90 (1.25-2.90)<br>HR 3.25 (1.82-2.79)	
Lin 2021	330	7.9	≥14%	DFS<br>OS	HR 1.44 (0.91-2.26)<br>HR 1.62 (0.89-2.92)	
Park 2021	5560	6.7	≥14%	DFS<br>OS	HR 2.45 (2.02-2.98<br>HR 3.33 (2.33-4.76)	Hazard ratios from univariate analyses
Parks 2021	299	NS		BCSS	NS	Study included solely women aged (≥ 70 years). The cut-off between high and low was H-score of 15<br>Difference between high and low Ki-67 was not significant
Qi 2021	1879	5.0	NS	BCSS<br>DMRS	HR 1.02 (1.00-1.03)<br>HR 1.01 (1.00-1.07)	Study included solely women treated with mastectomy and without adjuvant radiotherapy
Ugalde-Morales 2021	661	NS	≥20%	BCSS	HR 1.78 (0.90-3.54)	
Wang 2020	2812	5.0	>14%	RFS	HR 1.15 (0.73-1.81)	

Abbreviations: CI confidence interval; BCSS breast cancer–specific survival; BCSM breast cancer–specific mortality; CSH–LF cause–specific hazard for locoregional failure; CSH–DM cause–specific hazard for distant metastases; CNSM central nervous system metastases; DFS disease free survival; DR Distant recurrence; DMRS distant metastasis–free survival; HR hazard ratio; IBRFS ipsilateral breast recurrence-free survival; IDC invasive ductal carcinoma (IDC); ILC invasive lobular carcinoma; MFS metastasis free survival; NS not specified; OS overall survival; OM Overall mortality; RFP relapse–free period.

Supplementary Figures

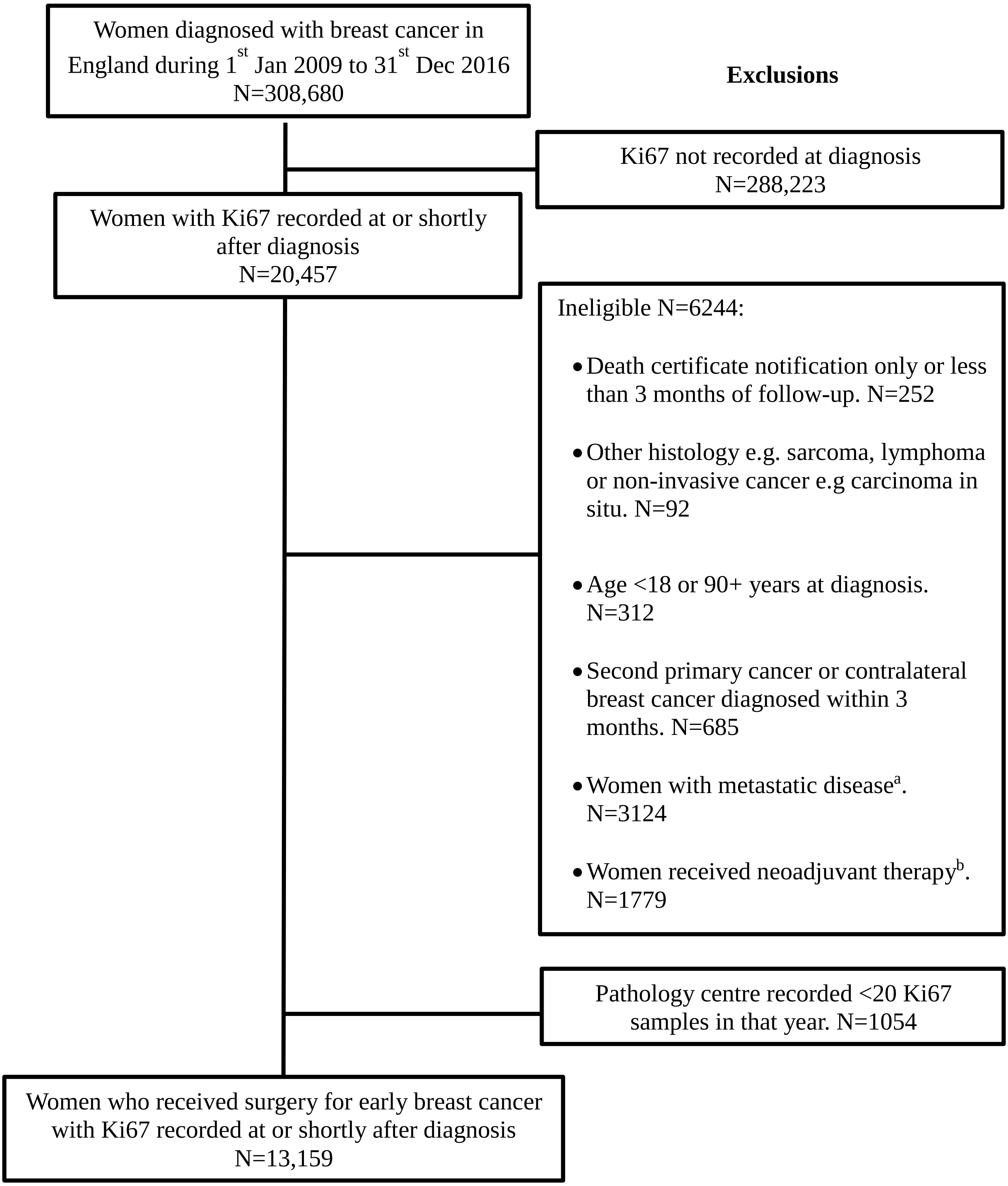

[a] Women with metastatic disease were identified as follows: no surgery recorded (N=1898) or with a record of metastatic disease within 3 months of breast cancer diagnosis, or a drug usually given for metastatic disease, or palliative radiotherapy within a year of breast cancer diagnosis (N=1226)

[b] Women recorded as receiving neoadjuvant therapy (chemotherapy, endocrine therapy, targeted therapy or radiotherapy) were excluded because comparable staging information was unavailable for them.

Supplementary Figure 1. Derivation of study population

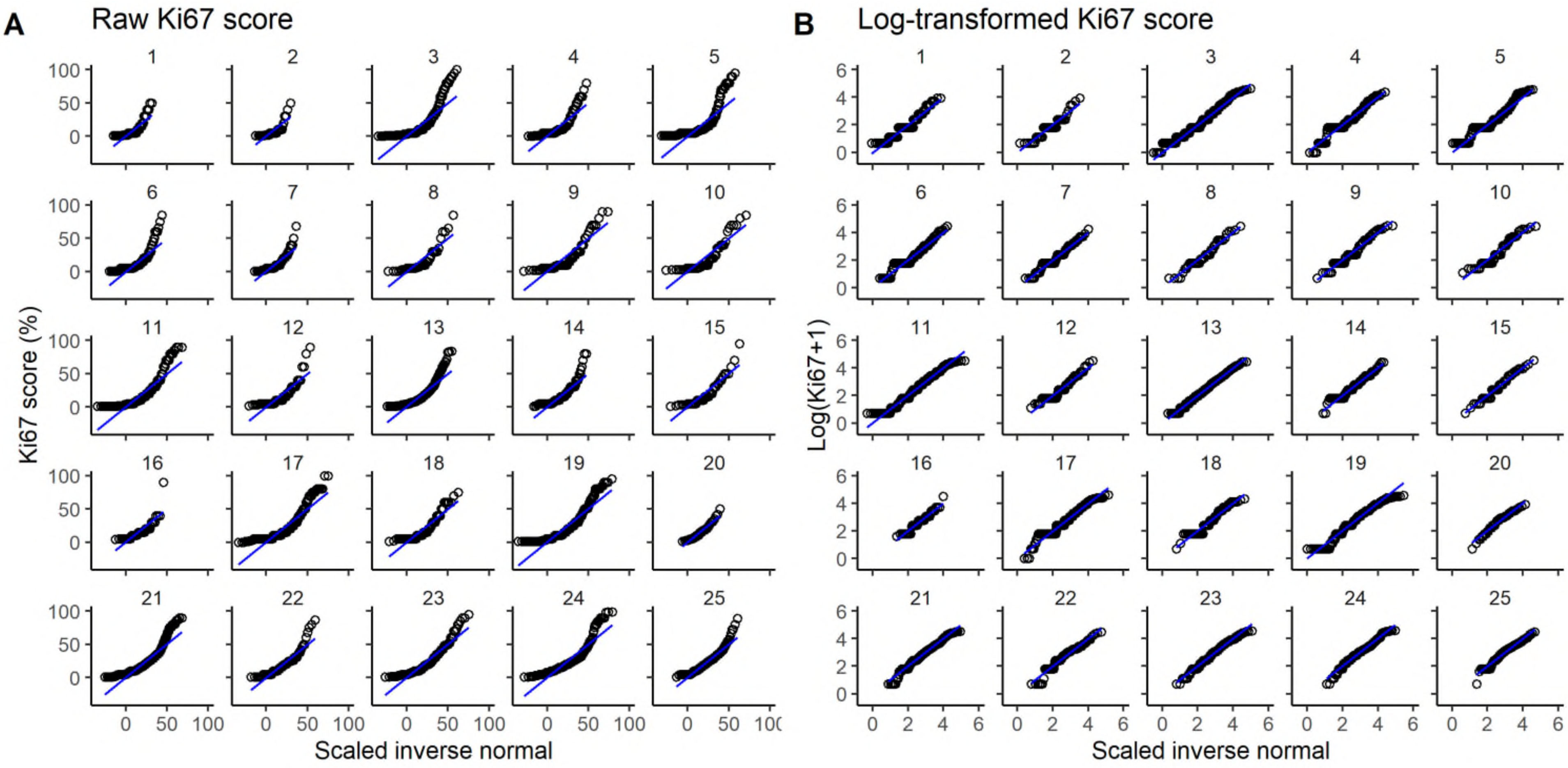

Supplementary Figure 2. Quantile-quantile plots of the A) raw and B) log-transformed Ki67 scores from the 25 pathology laboratories for 8212 women with ER-positive and HER2-negative breast cancer. See section D of Supplementary Text 1 for details.

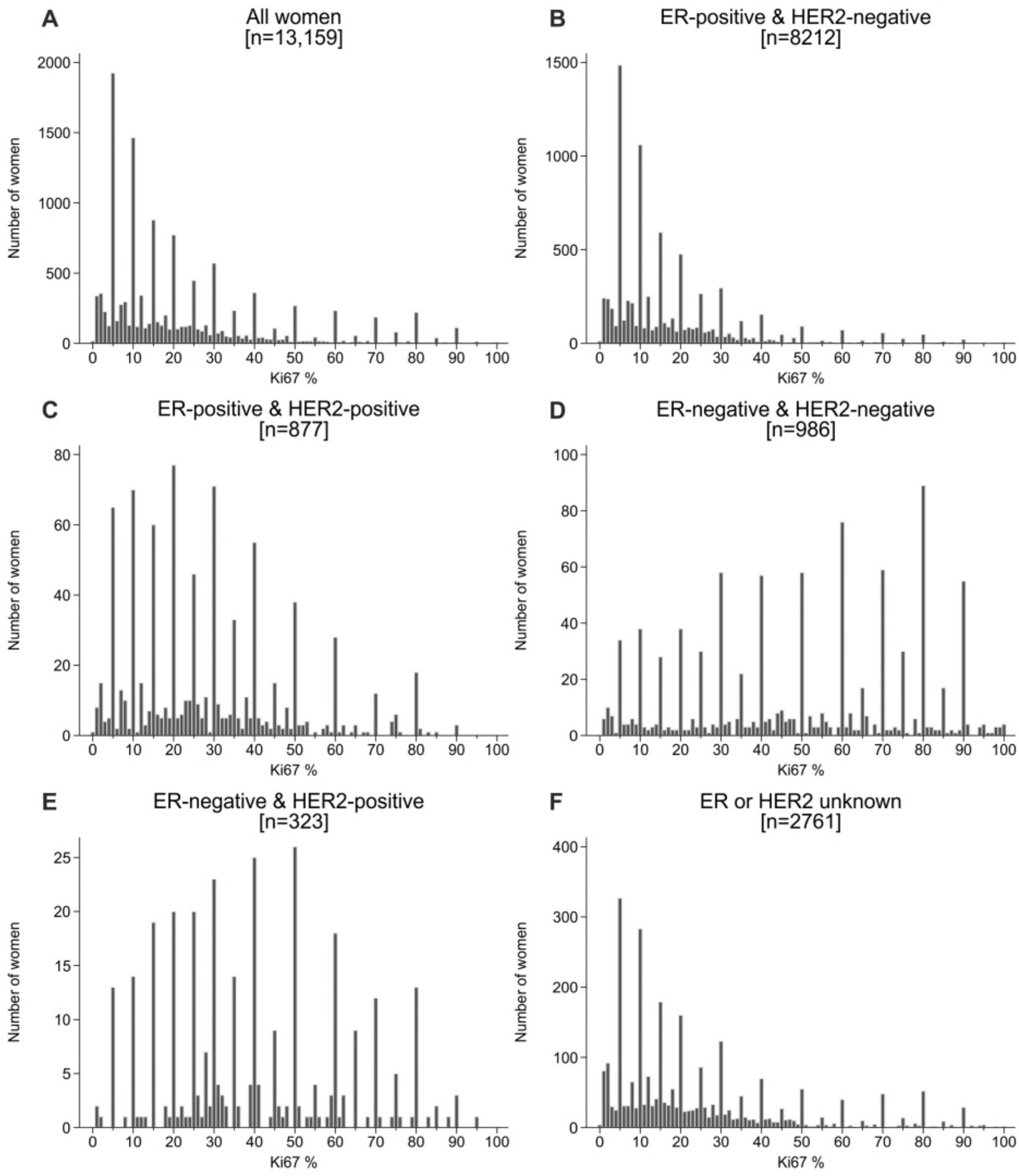

Supplementary Figure 3. Frequency distribution of Ki67 scores: (A) all women revealing digit preference in Ki67 scores; (B) – (F) subpopulations of women with different combinations of ER and HER2 status. Note that the scale of the y-axis differs between panels.

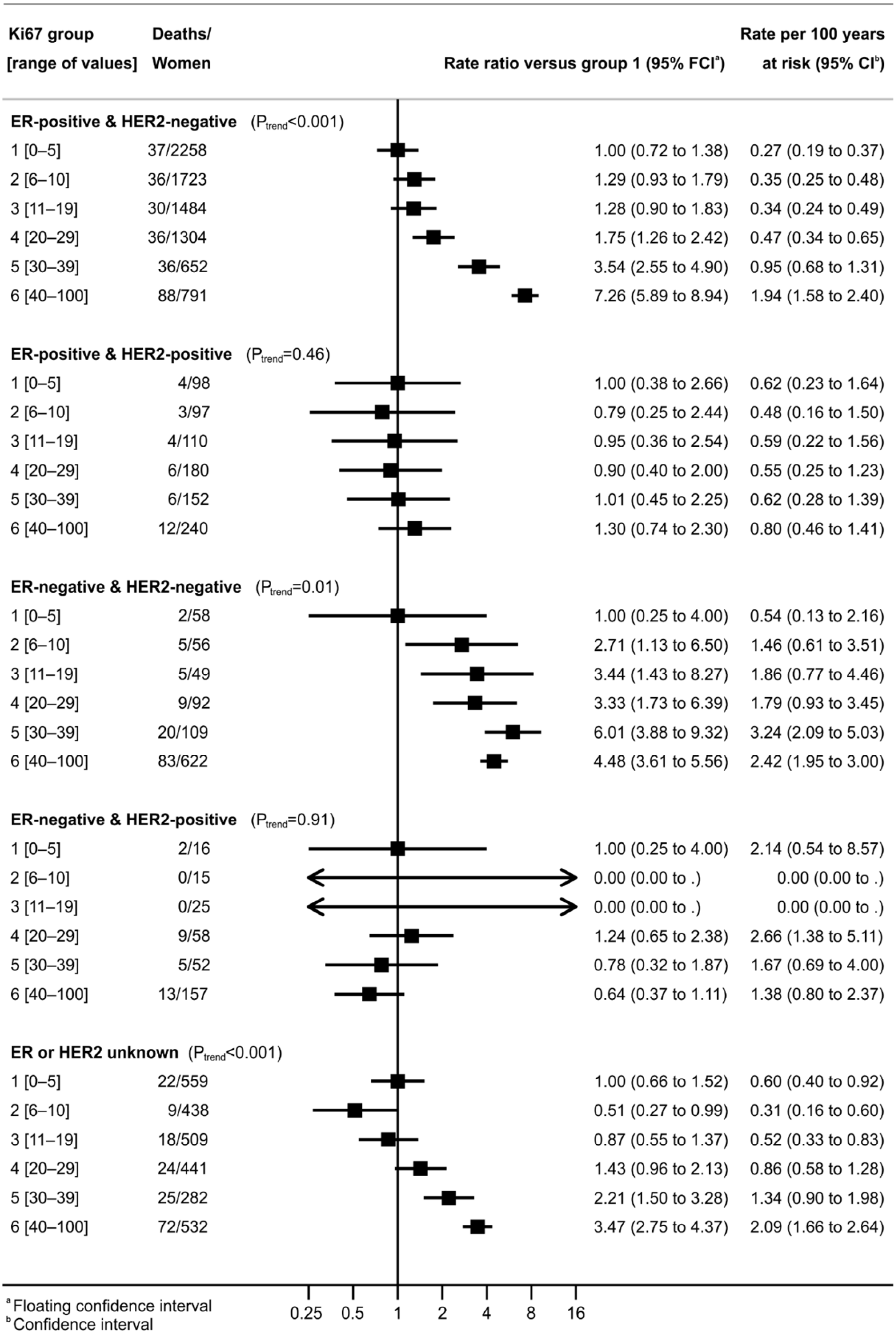

Supplementary Figure 4. Crude breast cancer mortality rates and rate ratios by Ki67 score (%), by ER and HER2 status

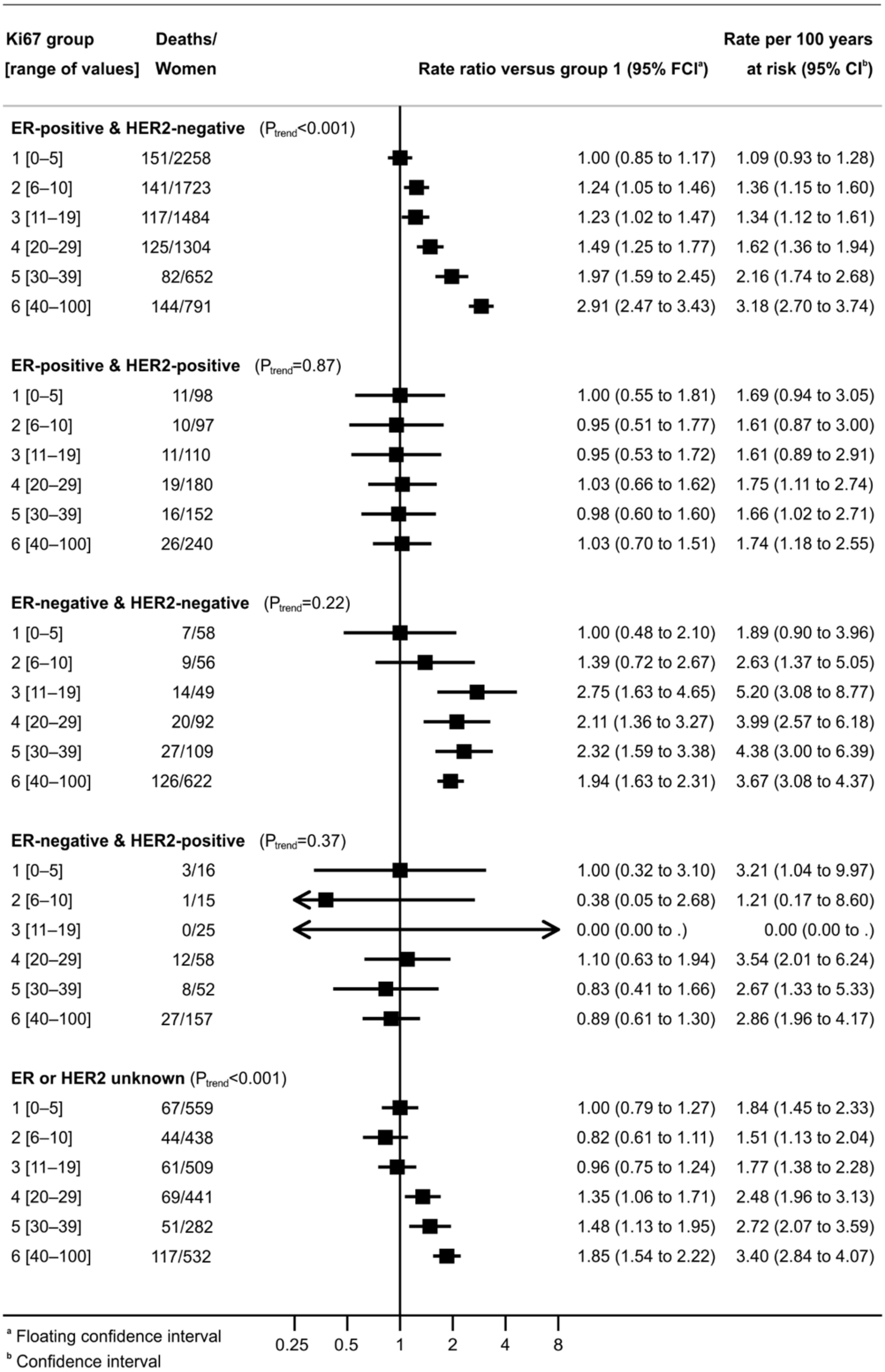

Supplementary Figure 5. Crude all-cause mortality rates and rate ratios by Ki67 score (%), by ER and HER2 status

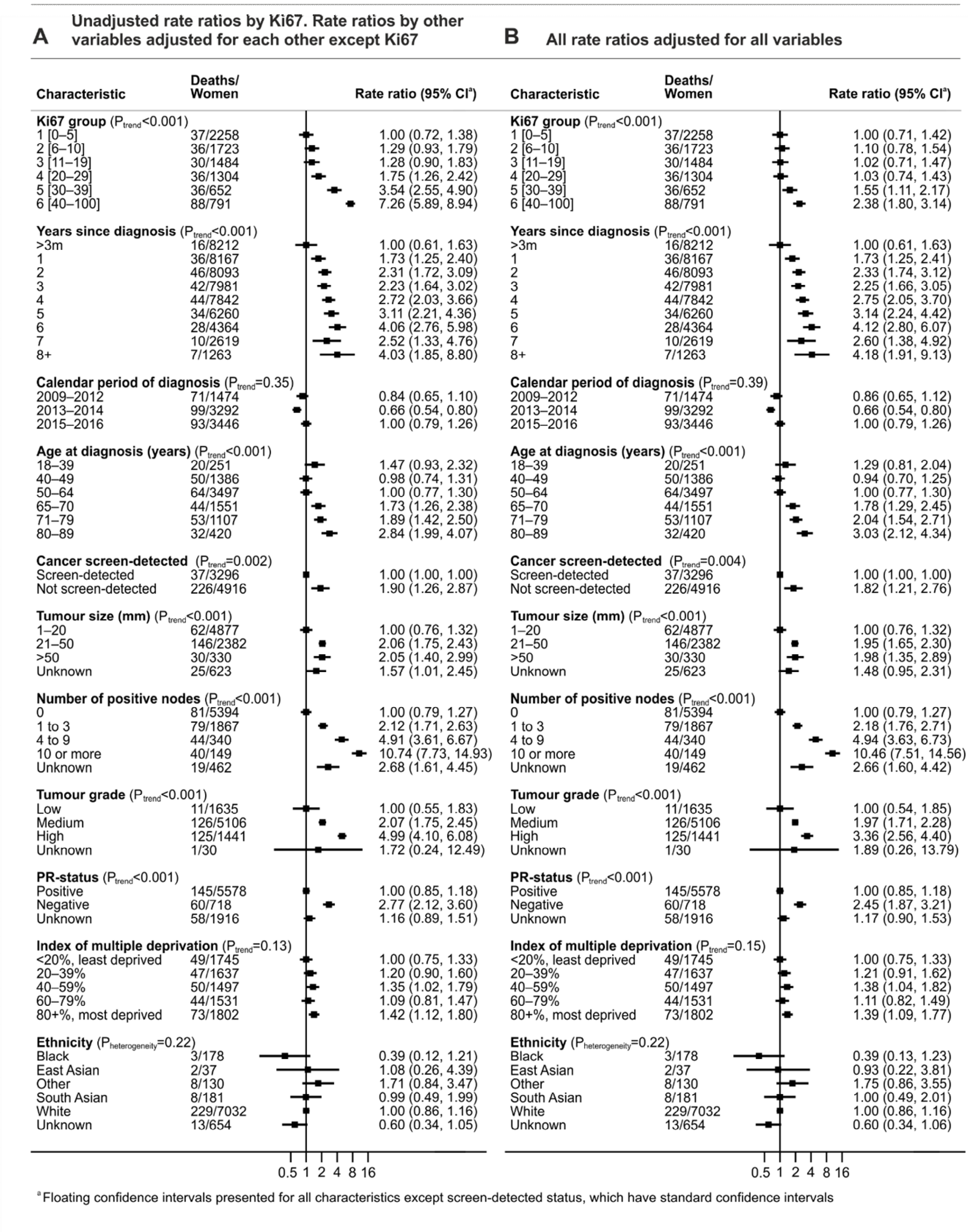

Supplementary Figure 6. Breast cancer mortality rate ratios for women diagnosed with ER-positive and HER2-negative early breast cancer by all available characteristics. On the left-hand side, Ki67 rate ratios are unadjusted, while the other characteristics are adjusted for every other characteristic (except Ki67) in the categories shown. On the right-hand side, for each characteristic, the rate ratios are adjusted for every other characteristic including Ki67 score.

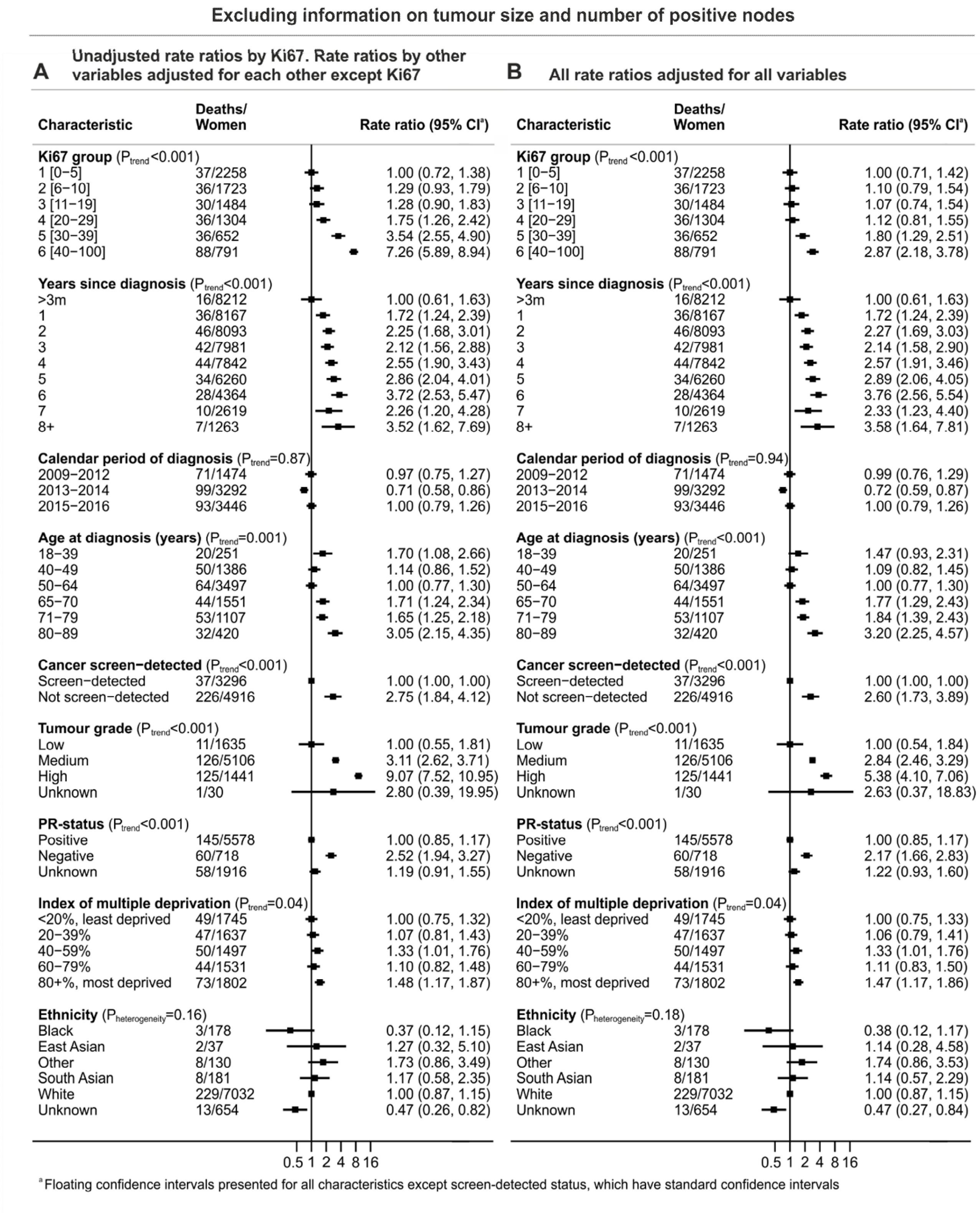

Supplementary Figure 7. Breast cancer mortality rate ratios for women diagnosed with ER-positive and HER2-negative early breast cancer with characteristics that would be available when considering patients for neoadjuvant therapy. On the left-hand side, Ki67 rate ratios are unadjusted, while the other characteristics are adjusted for every other characteristic (except Ki67) in the categories shown. On the right-hand side, for each characteristic, the rate ratios are adjusted for every other characteristic including Ki67 score.

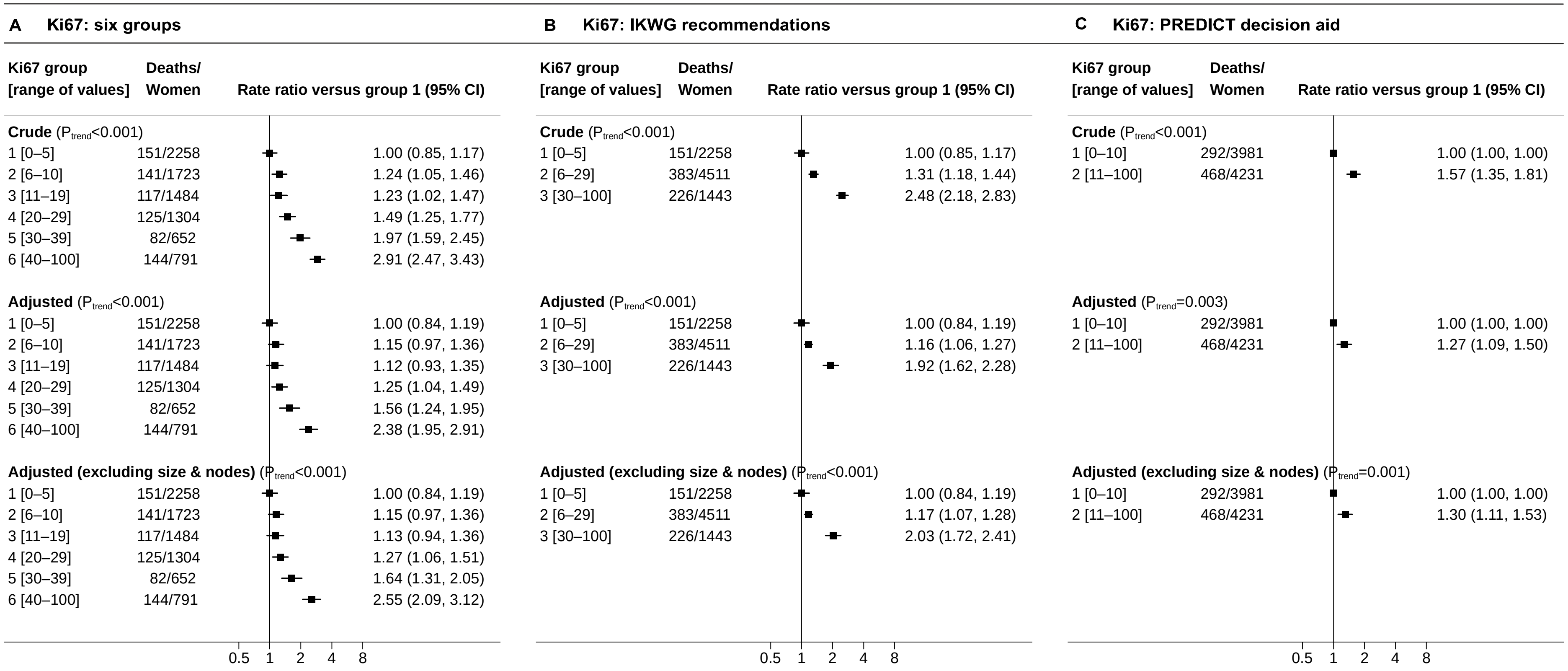

Supplementary Figure 8. All-cause mortality rate ratios in women with ER-positive and HER2-negative breast cancer by percentage Ki67 score. (A) Ki67 categorized in six groups, (B) Ki67 grouped according to the IKWG recommendations, (C) Ki67 grouped according to the PREDICT decision aid. Adjustment in middle row is for all variables shown in Table 1 (except chemotherapy) using the categories shown in Table 1. Adjustment in bottom row is similar but also omits tumor size and number of positive nodes.

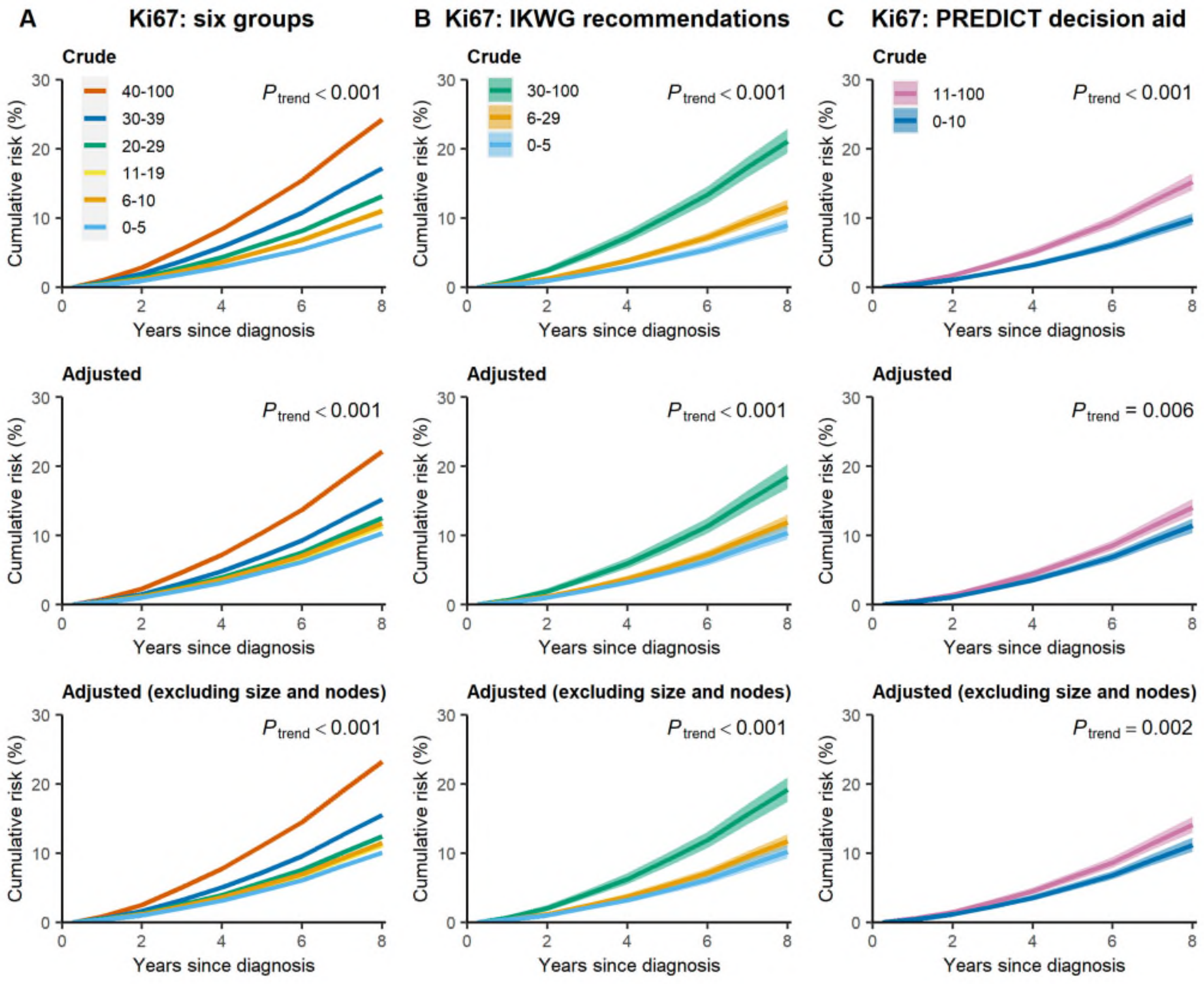

Supplementary Figure 9. Cumulative all-cause mortality risks in women with ER-positive and HER2-negative breast cancer by time since diagnosis and percentage Ki67 score. (A) Ki67 categorized in six groups, (B) Ki67 grouped according to the IKWG recommendations, (C) Ki67 grouped according to the PREDICT decision aid. Top row based on crude rates, middle row based on rates adjusted for all other variables shown in Table 1 (except chemotherapy), bottom row based on rates adjusted for all other variables in Table 1 except chemotherapy, tumor size and number of positive nodes. Shaded areas in (B) and (C) show 95% confidence intervals.

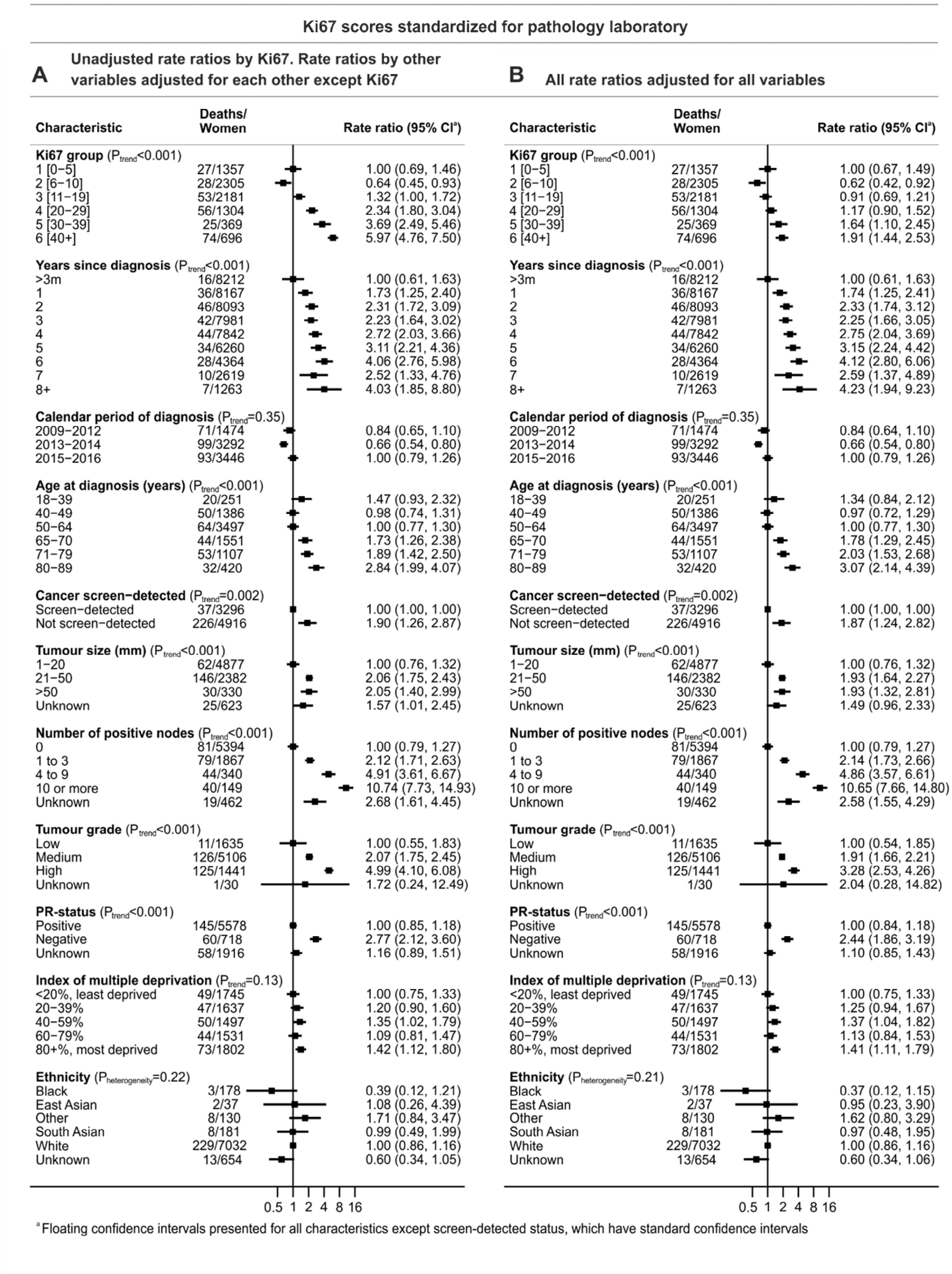

Supplementary Figure 10. Laboratory-standardized breast cancer mortality rate ratios for women diagnosed with ER-positive and HER2-negative early breast cancer. On the left-hand side, lab-standardized Ki67 rate ratios are unadjusted, while the other characteristics are adjusted for every other characteristic (except lab-standardized Ki67) in the categories shown. On the right-hand side, for each characteristic, the rate ratios are adjusted for every other characteristic including lab-standardized Ki67 score.

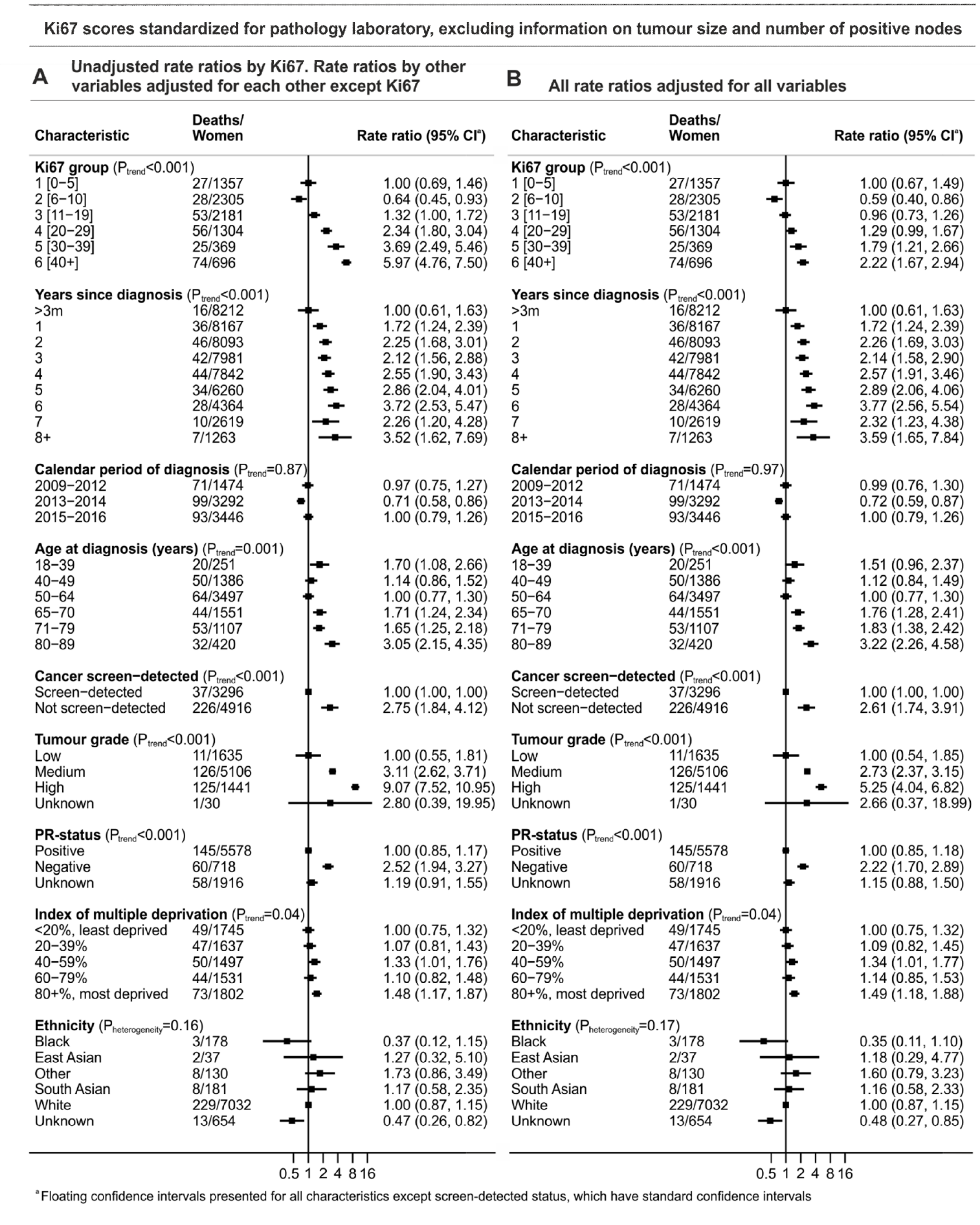

Supplementary Figure 11. Laboratory-standardized breast cancer mortality rate ratios for women diagnosed with ER-positive and HER2-negative early breast cancer with characteristics that would be available when considering patients for neoadjuvant therapy. On the left-hand side, lab-standardized Ki67 rate ratios are unadjusted, while the other characteristics are adjusted for every other characteristic (except lab-standardized Ki67) in the categories shown. On the right-hand side, for each characteristic, the rate ratios are adjusted for every other characteristic including lab-standardized Ki67 score.

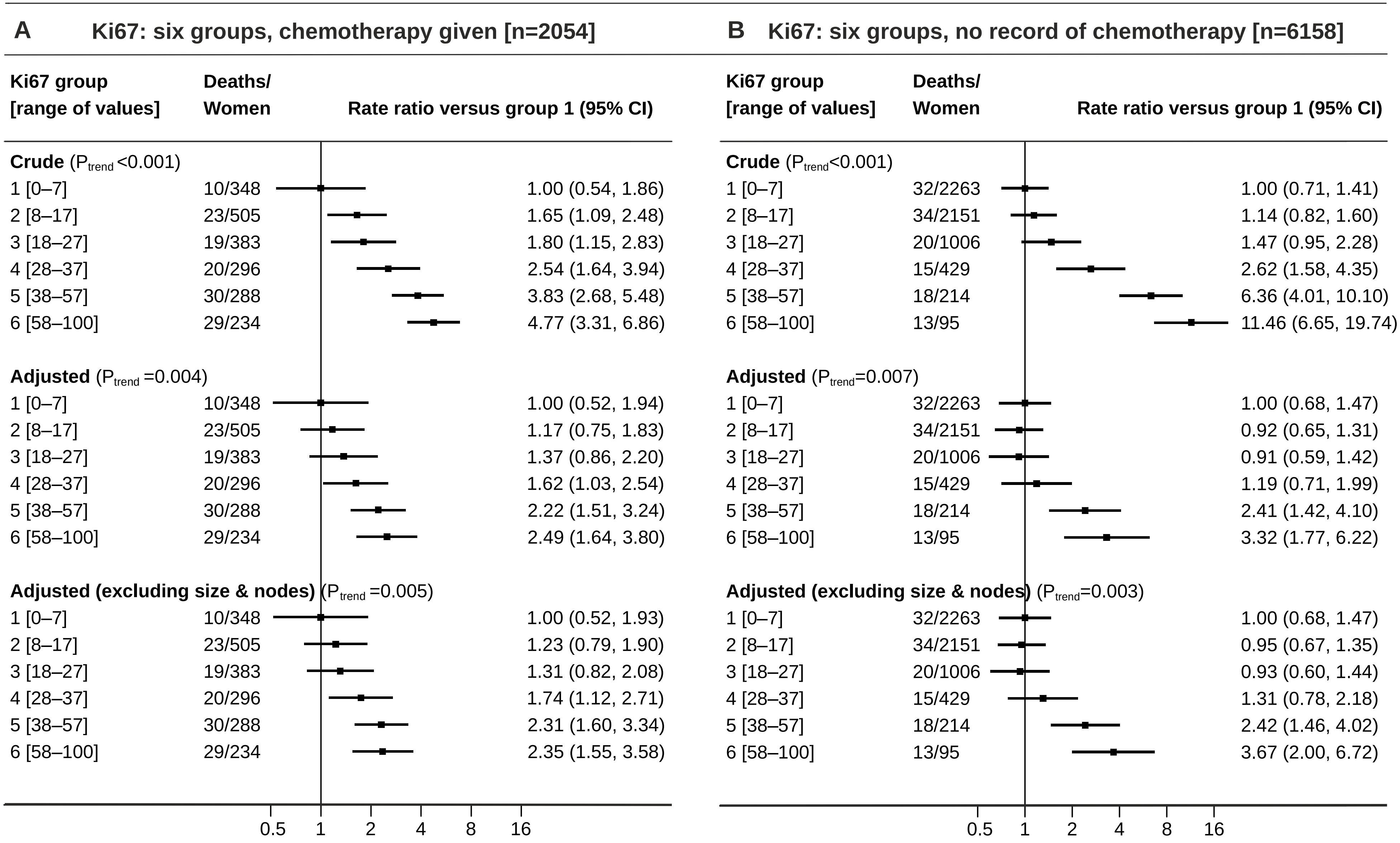

Supplementary Figure 12. Sensitivity analysis: Breast cancer mortality rate ratios by Ki67 score in women with (A) and without (B) a record of receiving chemotherapy treatment by Ki67 score classified into six groups avoiding the use of preferred digits as cut-points. Adjustment in middle panels is for all variables shown in Table 1 using the categories shown in Table 1. Adjustment in bottom panel is for all variables except tumor size and number of positive nodes.

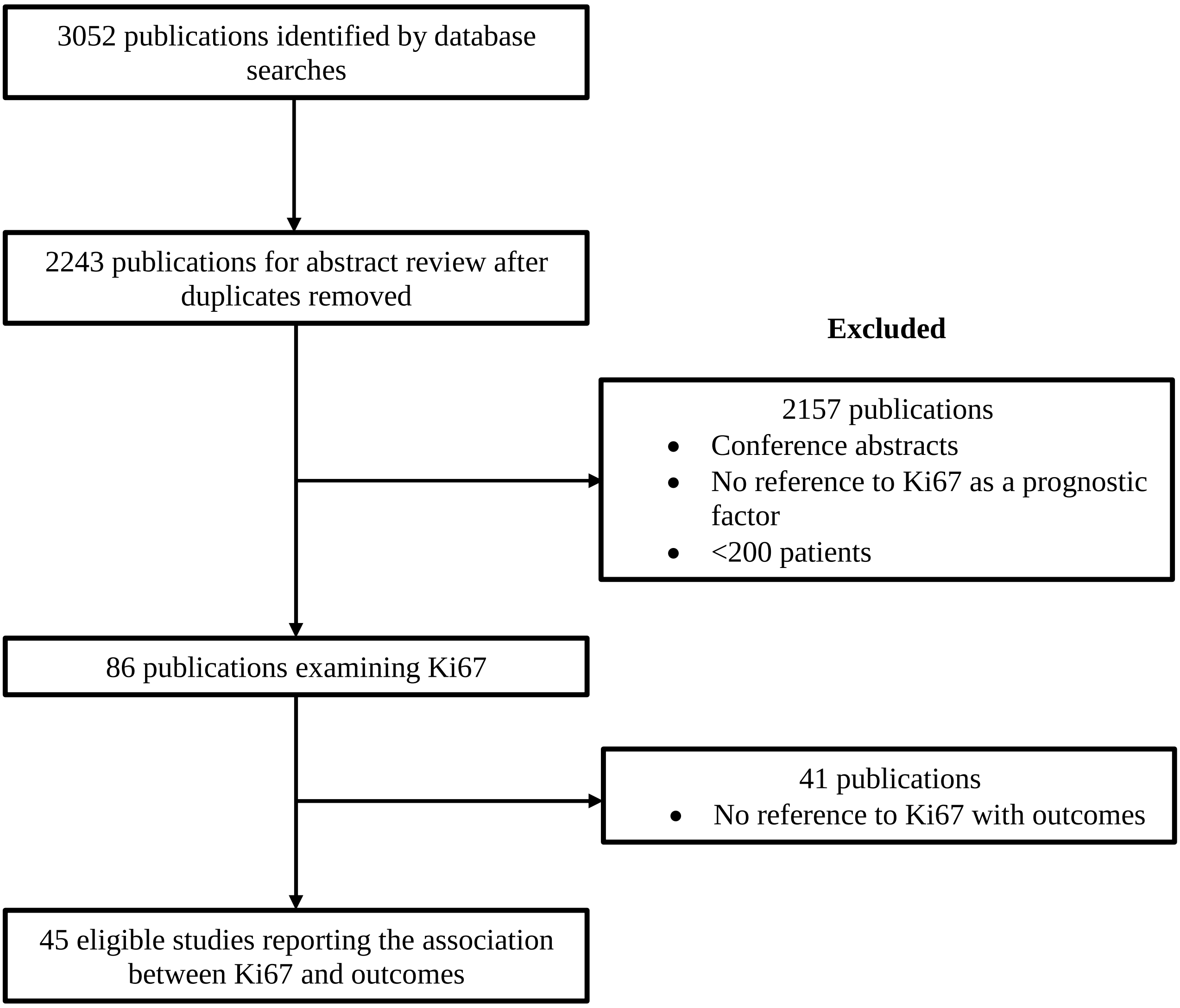

Supplementary Figure 13. The process of study identification for the literature review.

Medline and Embase were searched using the following search strategy: ((breast cancer.mp or Breast Neoplasms/) NOT metastatic.mp) AND (Cohort Studies/ OR (cohort adj (study or studies)).tw OR (observational adj (study or studies)).tw OR Cross-sectional studies/ OR Cohort analy$.tw OR prospective study.mp OR Prospective Studies/ OR real world studies.mp OR Retrospective Studies) AND (Ki-67 Antigen/ OR ki-67.pm)
